# Type I/type III IFN and related factors regulate JEV infection and BBB endothelial integrity

**DOI:** 10.1186/s12974-023-02891-x

**Published:** 2023-09-27

**Authors:** Ya-Ge Zhang, Hong-Xin Zhang, Hao-Wei Chen, Penghao Lv, Jie Su, Yan-Ru Chen, Zhen-Fang Fu, Min Cui

**Affiliations:** 1 State Key Laboratory of Agricultural Microbiology, College of Veterinary Medicine, Huazhong Agricultural University, Wuhan, 430070 Hubei China; 2 The Cooperative Innovation Center for Sustainable Pig Production, Huazhong Agricultural University, Wuhan, 430070 Hubei China; 3 Departments of Pathology, College of Veterinary Medicine, University of Georgia, Athens, GA USA; 4 Key Laboratory of Preventive Veterinary Medicine in Hubei Province, The Cooperative Innovation Center for Sustainable Pig Production, Wuhan, China

**Keywords:** Japanese encephalitis virus, Human brain microvascular endothelial cells, RNA-Seq, PRRs, IFNs, IFITs

## Abstract

**Background:**

Japanese encephalitis virus (JEV) remains a predominant cause of Japanese encephalitis (JE) globally. Its infection is usually accompanied by disrupted blood‒brain barrier (BBB) integrity and central nervous system (CNS) inflammation in a poorly understood pathogenesis. Productive JEV infection in brain microvascular endothelial cells (BMECs) is considered the initial event of the virus in penetrating the BBB. Type I/III IFN and related factors have been described as negative regulators in CNS inflammation, whereas their role in JE remains ambiguous.

**Methods:**

RNA-sequencing profiling (RNA-seq), real-time quantitative PCR, enzyme-linked immunosorbent assay, and Western blotting analysis were performed to analyze the gene and protein expression changes between mock- and JEV-infected hBMECs. Bioinformatic tools were used to cluster altered signaling pathway members during JEV infection. The shRNA-mediated immune factor-knockdown hBMECs and the in vitro transwell BBB model were utilized to explore the interrelation between immune factors, as well as between immune factors and BBB endothelial integrity.

**Results:**

RNA-Seq data of JEV-infected hBMECs identified 417, 1256, and 2748 differentially expressed genes (DEGs) at 12, 36, and 72 h post-infection (hpi), respectively. The altered genes clustered into distinct pathways in gene ontology (GO) terms and KEGG pathway enrichment analysis, including host antiviral immune defense and endothelial cell leakage. Further investigation revealed that pattern-recognition receptors (PRRs, including TLR3, RIG-I, and MDA5) sensed JEV and initiated IRF/IFN signaling. IFNs triggered the expression of interferon-induced proteins with tetratricopeptide repeats (IFITs) via the JAK/STAT pathway. Distinct PRRs exert different functions in barrier homeostasis, while treatment with IFN (IFN-β and IFN-λ1) in hBMECs stabilizes the endothelial barrier by alleviating exogenous destruction. Despite the complex interrelationship, IFITs are considered nonessential in the IFN-mediated maintenance of hBMEC barrier integrity.

**Conclusions:**

This research provided the first comprehensive description of the molecular mechanisms of host‒pathogen interplay in hBMECs responding to JEV invasion, in which type I/III IFN and related factors strongly correlated with regulating the hBMEC barrier and restricting JEV infection. This might help with developing an attractive therapeutic strategy in JE.

**Supplementary Information:**

The online version contains supplementary material available at 10.1186/s12974-023-02891-x.

## Background

Japanese encephalitis virus (JEV) is a critical member of the Flaviviridae family, whose infection severely threatens public health in Asia, Australia, and the Western Pacific [1, 2]. The occurrence of JEV infection generally leads to Japanese encephalitis (JE) in humans, which is characterized by a damaged blood‒brain barrier (BBB), CNS inflammatory response, persistent sustained neurologic or psychiatric sequelae in almost half of survivors, and an approximately 30% mortality rate [1, 3]. More seriously, children's cases of JEV infection have a high risk of developing severe JE. In addition, despite the approved vaccines, no specific therapy can be obtained under JE or other flavivirus infections.

Brain microvascular endothelial cells (BMECs), pericytes, and the end feet of astrocytes form the BBB, uniquely separating the brain parenchyma from blood circulation and restricting access to blood components, immune cells, and pathogens [4, 5]. BMECs are sealed together with tight junctions and serve as the primary structure to form the innermost lining of blood vessels, and preventing the entry of damaging substances [6, 7]. JEV-established infection in BMECs is thought to be the initial event and critical step of JEV disrupting the BBB [8–10]. Nevertheless, when JEV directly infects BMECs, no apparent disruption of the endothelial cell barrier appears [9]. Intriguingly, to extend the period of viral replication, JEV may interfere with the apoptotic pathway of transfected human brain microvascular endothelial cells (hBMECs) [8]. Aside from their well-characterized barrier function, BMECs can be immune activated to mount effective IFN induction and IFN-stimulated gene (ISG) expression as a response to viral infections, including human immunodeficiency virus (HIV), Zika virus (ZIKV), and influenza virus [11–13]. In restricting WNV transit, IFN-λ signaling promotes BMEC barrier tightening [14]. However, little is known about the relationship between host innate immune cytokines and BBB endothelial barrier integrity, and a more comprehensive understanding is needed.

The innate immune system serves as the front line of host defense against pathogens, which recognizes microorganisms, including viruses, via pattern-recognition receptors (PRRs) [15]. The interferon regulatory factor (IRF) is activated to induce IFN signaling, which triggers the expression of IFN-stimulated genes (ISGs) via the activated Janus kinase/signal transducer and activation of transcription (JAK/STAT) signaling pathway [16]. Nevertheless, a considerable portion of ISGs can also be induced in an IFN-independent manner [17]. Functionally, various ISGs, such as IFITs, can exert antiviral activity both in vitro and in vivo [18, 19]. In humans, IFITs consist of four members, namely, IFIT1 (also termed ISG56), IFIT2 (ISG54), IFIT3 (ISG60 or IFIT4), and IFIT5 (ISG58) [20]. In addition to antiviral immunity, PRRs also show distinct regulation of BBB endothelial barrier integrity [21]. The function of pleiotropic IFNs and induced ISGs includes maintaining endothelial barrier stability [22, 23]. In an in vitro BBB model, IFN-β treatment stabilizes BBB integrity [24, 25]. Moreover, the antiviral activity of IFN-λ1 is partly attributed to directly enhancing the properties of the endothelial barrier [26]. As an ISG, interferon-induced transmembrane protein 1 (IFITM1) has been identified as a tight junction protein that modifies the steady state of occludin to restrict HCV entry [23]. In addition, BBB permeability could be decreased by pretreatment with electroacupuncture ahead of cerebral ischemia‒reperfusion (I/R) injury in rats, along with an increase in antiviral-related genes (IFIT1 and IFIT3) [27]. However, during JEV infection, whether IFN and related factors (PRRs and IFITs) are involved in regulating hBMEC barrier integrity remains obscure.

To date, researchers have rarely focused on relative signaling events evoked in the response of BBB endothelial cells to JEV infection. Here, we have performed RNA-sequencing (RNA-seq) in JEV-infected hBMECs, with bioinformatic analysis and validation. Among the altered host genes and enriched signaling pathways, the PRRs (toll-like receptor 3, TLR3; retinoic acid-inducible gene-I, RIG-I; and melanoma differentiation-associated gene 5, MDA5)-IRF/IFNs (IFN-β and IFN-λ1)-JAK/STAT-IFITs signaling cascade was identified. Consistent with a previous report [28], IFIT1 restricts JEV replication, while other IFITs, including IFIT2, IFIT3, and IFIT5, were first characterized to exert antiviral activity in JEV infection. Then, we explored the function of IFN and related factors in modulating hBMEC barrier integrity. Knockdown of distinct PRRs in hBMECs differentially regulates endothelial barrier integrity. In addition to antiviral activity, treatment with rhIFN-β and rhIFN-λ1 in hBMECs protected the endothelial barrier from JEV-CM (JEV-infected astrocyte supernatants were mixed with an equal volume of fresh medium, which included inflammatory cytokines, data not shown)-caused disruption. However, the knockdown of IFITs in hBMECs did not abrogate the IFN-mediated maintenance of barrier integrity under treatment with JEV-CM. The study first mapped a comprehensive analysis of hBMECs responding to JEV infection through RNA-seq. Functionally, the regulatory role of type I/type III IFN and related factors in hBMEC barrier integrity during JEV infection was additionally provided.

## Methods

### Viruses and cell culture

The JEV P3 strain was derived from Beijing isolates previously preserved in our laboratory. Following the previously described protocol, JEV titration was detected using the baby hamster kidney fibroblast cell line (BHK-21) [29]. The heat-inactivated JEV P3 (heated-JEV P3) was generated by incubating at 94 °C for 15 min [30]. The hBMEC cell line was kindly provided by Prof. Xiangru Wang (Huazhong Agricultural University, Wuhan, China) and routinely cultured as previously described [31]. Cells were washed three times and then starved in serum-free medium for 12–16 h before further experiments. BHK-21, HEK-293T, and Vero cells were cultured in Dulbecco’s modified Eagle’s medium (DMEM) (Gibco) supplemented with 10% heat-inactivated FBS and penicillin/streptomycin (100 U/ml).

### Reagents, antibodies, and inhibitors

Human IFN-β recombinant protein (rhIFN-β) and human IFN-λ1 recombinant protein (rhIFN-λ1) were purchased from Abbkine (Wuhan, China) and MedChemExpress (Shanghai, China). The Cell Counting Kit (CCK-8) was obtained from Biosharp (Anhui, China). Pyridone 6 (JAK inhibitor I) (A13457) was purchased from Adooq Biosciences (Irvine, CA, USA). Antibodies used for Western blotting, anti-phospho-STAT1 (S727), anti-STAT1, and anti-IFI35 antibodies (all rabbit polyclonal antibodies), were obtained from Abmart (Shanghai, China). Anti-IFIT2, anti-TLR3, anti-IRF7, anti-IRF3, anti-USP18, and anti-ISG15 antibodies (all rabbit) were purchased from ABclonal (Wuhan, Hubei, China). Anti-JAK1, anti-JAK2, anti-phospho-JAK1, and anti-phospho-JAK2 antibodies (all rabbit) were purchased from Abcam (Cambridge, MA, USA). Anti-MDA5 (IFIH1) and anti phospho-IRF3 were purchased from Cell Signaling Technology (Danvers, MA, USA). Anti-RIG-I (DDX58), anti-IFIT1, anti-IFIT3, anti-IFIT5, anti-IRF1, anti-IFITM1, and anti-β-actin antibodies were purchased from Proteintech (Chicago, IL, USA), and the antibodies used are listed in Additional file 7: Table S7. The monoclonal antibody against the JEV envelope (E) was kindly provided by Shengbo Cao (Huazhong Agricultural University, Wuhan, China). For immunofluorescence, rabbit anti-FLAG antibody was purchased from Proteintech.

### RNA extraction, cDNA library construction, and RNA-Seq

A total of 18 hBMEC cell samples were collected at three time points (12, 36, and 72 hpi). At each time point, three samples were drawn from the negative control group, and three were drawn from the JEV infection group. According to the manufacturer’s protocol (Invitrogen, Grand Island, NY, USA), total RNA was extracted using TRIzol® Reagent and stored at − 80 °C until further use. As previously described [32], 1 μg RNA per sample was utilized as input material for the RNA sample preparations for library construction.

### Reverse transcription and real-time PCR

Whole-cell RNA was extracted using TRIzol reagent (Invitrogen, Grand Island, NY) and reverse transcribed into cDNA. Quantitative real-time PCR (RT-qPCR) was performed with SYBR Green 2 × mix (Invitrogen). The data were normalized to β-actin levels and calculated by the 2^−ΔΔCT^ method except for the JEV-C gene, as described previously [33]. By utilizing the pcDNA3.0-HA/JEV-C gene plasmid as a template, a standard curve was constructed for the quantification of the JEV-C gene copy number as per our previously described procedures [33]. In each RT-qPCR experiment, at least three replications were conducted, and the primers used are listed in Additional file 1: Table S1.

### Construction of lentivirus containing target genes and hBMEC cell lines

As previously described, shRNAs targeting TLR3, RIG-I, MDA5, IFIT1, IFIT2, IFIT3, and IFIT5 were designed (shown in Additional file 1: Table S2), and hBMEC cell lines were constructed [34].

### Immunofluorescence (IF) and Western blotting (WB) analysis

The cells were cultured in glass-bottomed dishes (diameter 35 mm) or well plates for the specified treatment and further experiments. Both immunofluorescence assays and Western blotting analysis were performed as described previously [33, 35].

### ELISA

ELISA kits for IFN-β and IFN-λ1 were purchased from 4A Biotech Co., Ltd. hBMEC culture supernatants were collected at different time points, and the protein levels were determined following previous descriptions [36, 37].

### Cell viability assay

For the cell viability assay, both the CCK-8 assay and Annexin V and propidium iodide (AnnV/PI) staining were performed as previously described [12, 33].

### Transendothelial electrical resistance

For the coculture BBB model, hBEMCs were plated on the upper part of transwell inserts (Corning Costar, USA; 0.4 μm membrane) in a 12-well plate containing confluent human astrocytes as previously described [38]. When the two cell lines reached confluence, astrocytes were infected with JEV or heated JEV, and at different time points, the transendothelial electrical resistance (TEER) was measured with a Millicell ERS ohmmeter (Millipore, Billerica, MA) as described in a previous study [39]. In the established hBMEC monolayer BBB model, briefly, cell monolayers were grown on the upper part of transwell inserts in a 12-well plate until confluent. After treatment for the appropriate time period, the TEER was measured at different time points as described previously [39].

### Statistical analysis

The data are expressed as the means ± SEMs unless otherwise stated. Student’s *t* test and one-way analysis of variance (ANOVA) were applied to analyze the statistical significance of the differences by using GraphPad Prism (v7.0; GraphPad, La Jolla, CA, USA). A value of *p* < 0.05 (*) was considered statistically significant, and *p* < 0.01 (**), *p* < 0.001 (***), and *p* < 0.0001 (****) indicated extremely significant differences.

## Results

### Characteristics of hBMECs infected with JEV

**Fig. 1:**
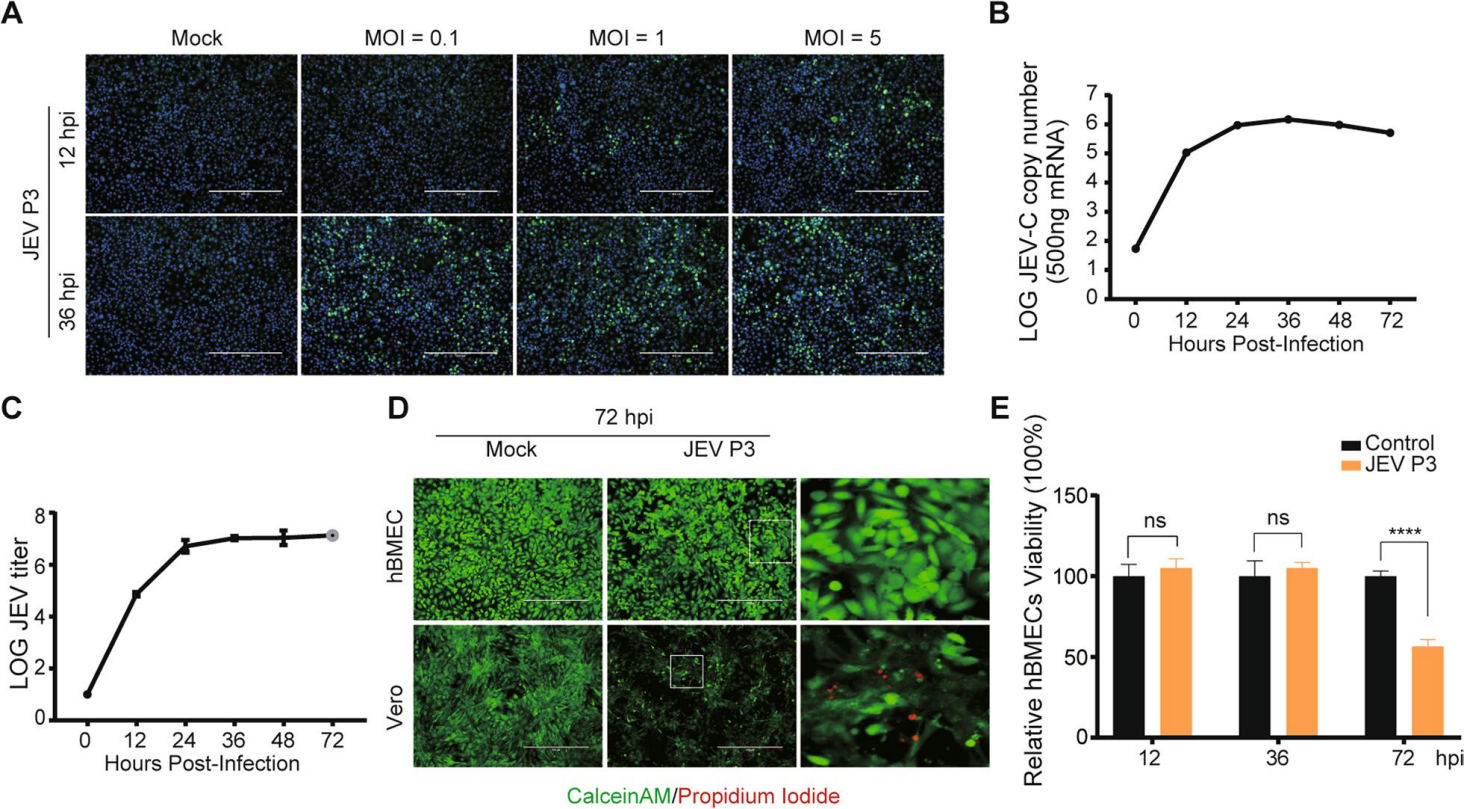
JEV infection of hBMECs. **A** hBMECs were infected with JEV P3 at MOIs of 0.1, 1, and 5. At 12 and 36 h post-infection (hpi), JEV-E was detected by indirect immunofluorescence (green), and nuclei are shown by Hoechst (blue) staining. **B**, **C** JEV RNA levels were assayed by quantitative real-time PCR (RT-qPCR) (B), and the titers were determined by PFU assay (**C**). **D** Vero or hBMECs were infected with JEV P3 for 72 h and then costained with calcein-AM (green [live cells])/propidium iodide (red [dead cells]) for another 45 min. Images were captured by fluorescence microscopy. **E** hBMECs were infected with JEV P3 for 72 h, and then cell viability was analyzed by CCK8 assay. Data are represented as the mean values ± SEMs from three independent experiments. ****p < 0.0001; ns: not significant

To characterize the impact of JEV infection on BBB endothelial cells, hBMECs were infected with the JEV P3 strain at multiplicities of infection (MOIs) of 0.1, 1, and 5, and infectivity was assessed by indirect immunofluorescence assay. Based on the obtained results (Fig. 1A), an MOI of 1 was identified as the inoculum dose for further experiments. The RNA levels of JEV in hBMECs and viral titers in the supernatants increased from 12 to 48 hpi but slightly decreased at 72 hpi (Fig. 1B, C). The JEV-induced cytopathic effect (CPE) in hBMECs is inconspicuous but quite distinct from that established in Vero cells [40]. Then, the PI/calcein-AM uptake assay was performed to compare the survival of hBMECs and Vero cells following JEV infection. As illustrated, nearly all hBMECs were PI-negative and viable. In contrast, more than 50% of Vero cells remained PI-positive (dead) at 72 hpi of JEV infection (Fig. 1D). Given the lack of CPE in JEV-infected hBMECs, a CCK-8 assay was performed to determine cell viability. Compared with mock infection, there was no significant change in cell viability at 12 and 36 hpi, but it was significantly lowered at 72 hpi (Fig. 1E). These findings confirmed that JEV could not cause CPE but decreased cell viability at the late phase of infection in hBMECs.

### Identification of differentially expressed genes (DEGs)

**Fig. 2:**
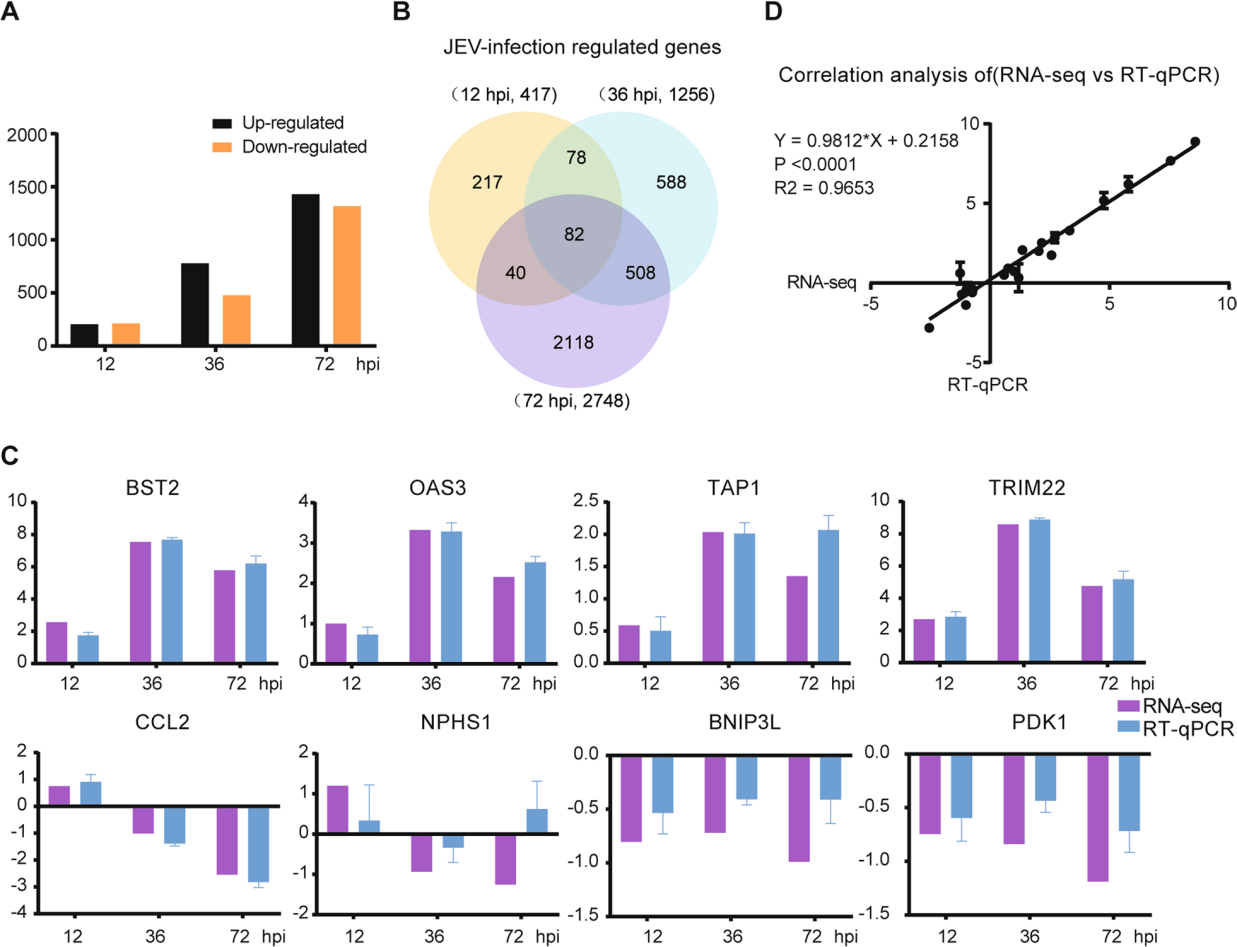
Schematic view and validation of transcriptome data in JEV-infected hBMECs. **A** The number of DEGs at 12, 36, and 72 hpi in hBMECs infected with JEV P3; **B** Venn diagram showing the distributions of unique and codifferentially expressed DEGs; **C** RT-qPCR was performed to validate the expression of eight representative genes. **D** GraphPad software 6.0 (San Diego, CA) was used to perform correlation analysis between RT-qPCR (y-axis) and the RNA-Seq platform (x-axis). Data are represented as the mean values ± SEMs from three independent experiments

To map the comprehensive information of JEV-infected hBMECs, RNA-Seq was carried out at 12, 36, and 72 hpi, and the raw reads were quality controlled to ensure that all data met the criteria for the whole transcriptomic analysis. Principal component analysis (PCA) illustrated group differences and consistency (Additional file 2: Fig. S1). In Additional file 3: Table S3, genes with 1.5-fold or more significant changes were defined as DEGs (P < 0.05). A total of 4151 DEGs were identified, and 417, 1256, and 2748 genes were identified in the following comparison groups: mock vs. hBMECs infected with JEV for 12 h (JEV12h), mock vs. JEV36h, and mock vs. JEV72h, respectively (Additional file 4: Fig. S2A–C). As shown, the number of DEGs increased with JEV infection time, with 205, 779, and 1430 genes upregulated and 212, 477, and 1318 genes downregulated at 12, 36, and 72 hpi, respectively (Fig. 2A). Next, Venn diagrams were constructed to identify either continuous DEGs (genes that were differentially expressed at all time points) or specific DEGs at each time point, which revealed the presence of only 82 DEGs at three time points, indicating a time-related alteration in gene expression (Fig. 2B). Then, RT-qPCR was performed on eight genes selected from continuous DEGs to validate the repeatability and reality of RNA-sequencing (RNA-seq). The differential expression trends of these genes were confirmed in all the groups, which were consistent with these changes based on RNA-seq results (Fig. 2C). According to the scatter plotting log2-fold changes with the 0.988 correlation coefficient (R^2^), it can be concluded that a strong positive correlation exists between the RT-qPCR and RNA-Seq data (Fig. 2D).

### GO and KEGG enrichment analysis

Enrichment analysis of DEGs is a critical way to screen the essential genes of hBMECs responding to JEV infection, which may help us to find new insights. Therefore, GO and KEGG enrichment analyses were performed at 12, 36, and 72 hpi. The GO terms classified the functions of all DEGs into biological process (BP), cellular component (CC), and mechanism functions (MF). BP had the most enriched cluster at each of the three time points, in which the DEGs were mainly involved in defense responses to the virus, innate immune responses, and viral regulation (Additional file 4: Fig. S3A, C, E). The details of the GO enrichment analysis are shown in Additional file 5: Table S4.

The top 30 enriched pathways in DEGs across all three time points were represented based on KEGG pathway enrichment analysis, which primarily concentrated on host innate immune-relevant pathways, including the RIG-I-like receptor signaling pathway, JAK–STAT signaling pathway, and Toll-like receptor signaling pathway (Additional file 4: Fig. S3B, D, F). The details of the KEGG enrichment analysis are shown in Additional file 5: Table S5.

### Global transcriptional changes in JEV-infected hBMECs

Based on the GO and KEGG databases, the global transcriptional responses of hBMECs to JEV infection were analyzed. The prominent DEGs selected are presented in Table S6, with complete data files deposited in the NCBI SRA database under accession number PRJNA936274. The upregulation of ISG15, ISG20, ISG15 family ligases (HERC5/6), and ISG15 proteases (USP18) also appeared upon infection, which has been reported to participate in host antiviral immunity [41–44]. The canonical proinflammatory cytokines, including IL-1β/IL-6, did not increase after infection (Additional file 5: Table S6), indicating that hBMECs might not be the primary source of inflammatory cytokines in JEV-induced CNS inflammation. Of note, elevated expression of interferon-inducible protein 35 (IFI35) was observed in JEV-infected hBMECs (Additional file 6: Fig. S4A, B), which has been reported to prompt various viral infections by negatively regulating RIG-I signaling [45, 46]. Nevertheless, overexpressing IFI35 in HEK-293T cells failed to accelerate JEV replication (Additional file 6: Fig. S4C). Additionally, genes related to BBB regulation, including CXCL10, CCL5, and ICAM2, were also induced during JEV infection (Additional file 5: Table S6) [47–49]. The DEGs considered critical were identified at the transcriptional or translational levels, most of which displayed increased expression (Additional file 6: Fig. S5A, B).

### Poly(I: C) treatment or JEV infection induces IFIT expression, which exerts antiviral activity

**Fig. 3:**
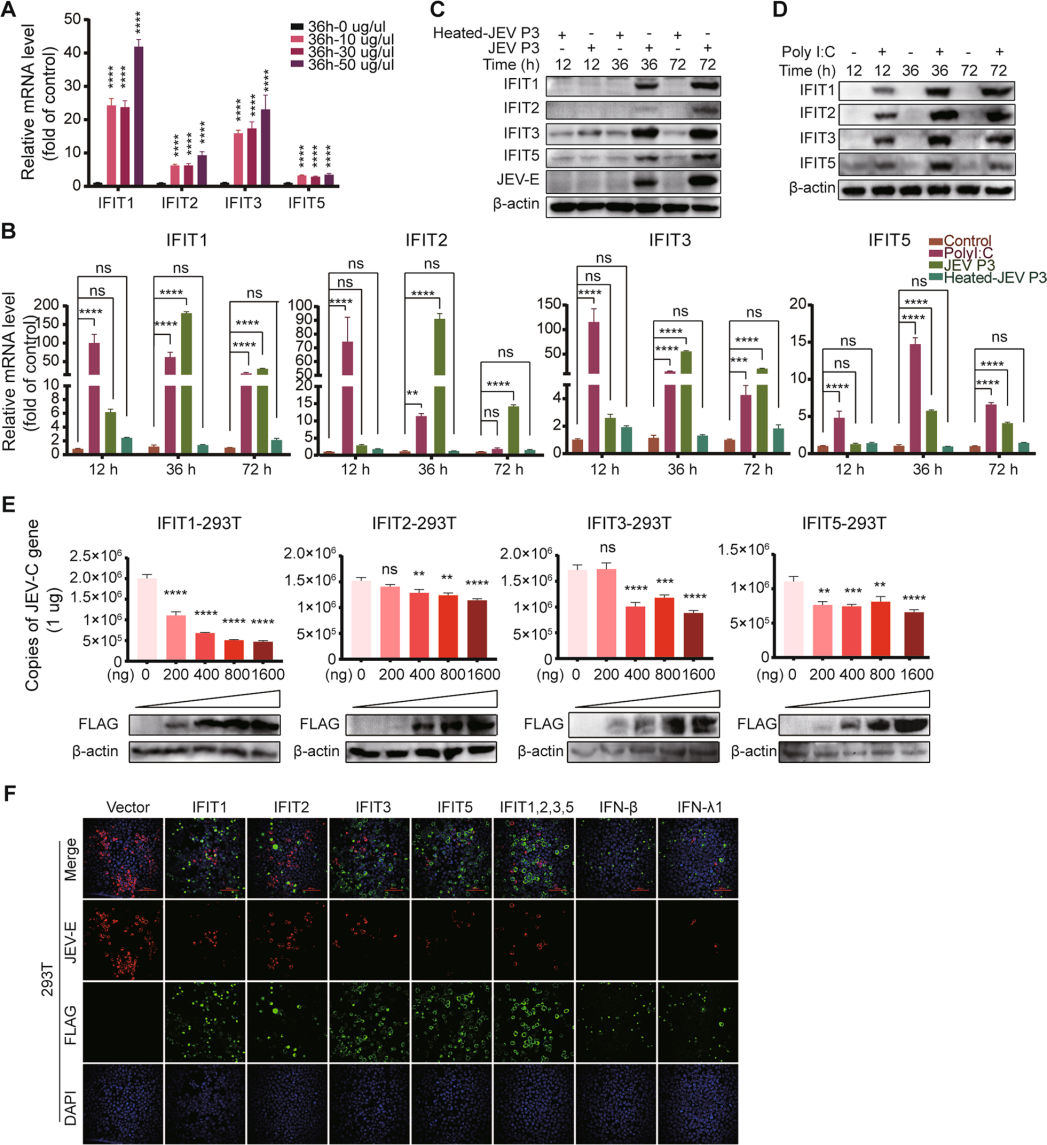
Induction and antiviral activity of IFITs. **A** hBMECs were treated with poly I:C at different concentrations, and IFIT mRNA expression was measured. **B**–**D** hBMECs were infected with JEV P3 or heated-inactivated JEV P3 (heated-JEV P3, 94 °C, 15 min) or treated with poly I: C for different time points, and IFIT expression was measured by performing RT-qPCR (**B**) and Western blotting (**C**, **D**). **E**, **F** HEK-293T cells were transfected with IFIT1, IFIT2, IFIT3, and IFIT5 expression plasmids or empty vector plasmids at the indicated doses following infection with JEV P3. Then, the JEV-C gene was analyzed by RT-qPCR (**E**), and the JEV-E protein was measured by performing an immunofluorescence assay (**F**). Data are represented as the mean values ± SEMs from three independent experiments. **p < 0.01; ****p < 0.0001; ns: not significant

Our RNA-seq data revealed the significant upregulation of IFITs, including IFIT1, IFIT2, IFIT3, and IFIT5 (Additional file 5: Table S6), in hBMECs responding to JEV infection. As an inducer of IFIT expression [35], poly(I: C) was first employed in concentration gradients in hBMECs, and a dramatic increase in IFIT mRNA was observed (Fig. 3A). To further confirm the RNA-seq data, RT-qPCR was performed, and all the IFITs showed an upward trend in transcription levels under poly(I: C) treatment or JEV infection (Fig. 3B). With specific antibodies, accelerated expression of IFITs in translation levels appeared at 36 and 72 h post-JEV infection and at each time point under poly(I: C) treatment but not post-heated JEV infection in comparison with the uninfected control group (Fig. 3C, D). The results demonstrated elevated expression of IFITs in hBMECs after both treatment with poly(I: C) and infection with JEV.

It has been reported that IFIT1 was proven to exert antiviral responses in JEV infection [28], whereas other IFITs remain unknown. Therefore, an overexpression assay was performed. Human IFITs (hIFIT1, hIFIT2, hIFIT3, hIFIT5) were transfected into HEK-293T cells at the corresponding doses, followed by JEV infection for 48 h. The overexpression of all IFITs showed a dose-dependent inhibitory effect at the viral RNA (JEV-C) level (Fig. 3E) compared to the mock control (empty vector). At the protein level, consistent results were acquired in the immunofluorescence assay. The number of JEV-E-positive cells showed significant decreases in each of the IFIT-, IFN-β-, and IFN-λ1-overexpressing groups (Fig. 3F). These findings demonstrated that all human IFITs could exert antiviral activity against JEV.

### Identified PRRs are associated with hBMEC barrier integrity and the induction of IFITs

**Fig. 4:**
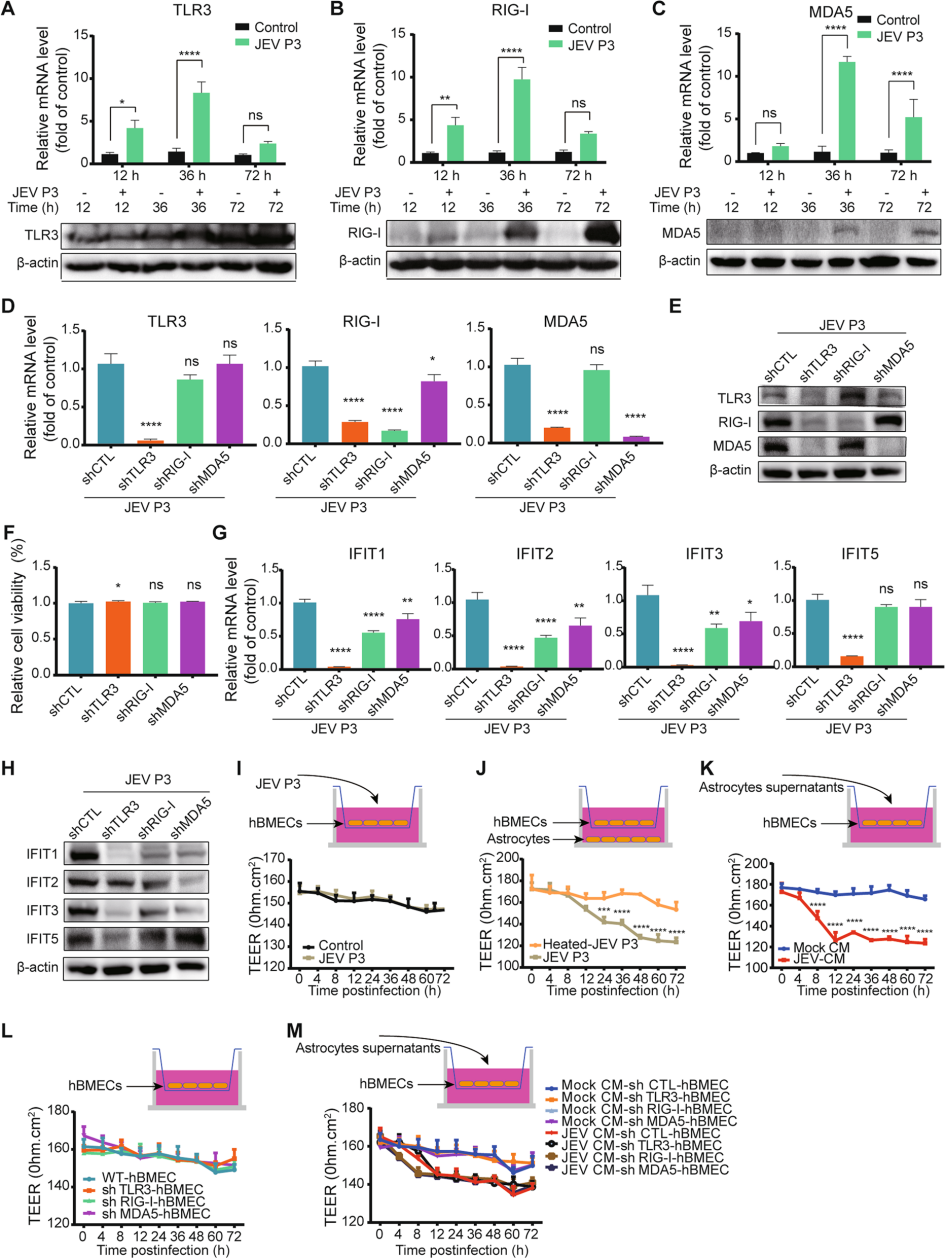
The role of PRRs in IFIT induction and hBMEC barrier maintenance. **A**–**C** hBMECs were infected with JEV P3 for different time points, and the mRNA and protein levels of TLR3 (**A**), RIG-I (**B**), and MDA5 (**C**) were measured. **D**, **E** PRR-knockdown hBMECs were infected with JEV P3, and the expression of TLR3, RIG-I, and MDA5 was assessed by RT-qPCR (**D**) and Western blotting (**E**). **F** PRR-knockdown hBMECs were serum starved after reaching confluence, and cell viability was measured. **G**, **H** Expression of IFITs was assessed by RT-qPCR (**G**) and Western blotting (**H**) in PRR-knockdown hBMECs. **I** Confluent monolayers of hBMECs in the upper chamber of the transwell plate were infected with JEV P3, and the TEER (upper graph) was measured at the indicated times. **J** hBMECs were seeded onto the upper chamber, cultured astrocytes infected with JEV P3 or heated-JEV P3 were placed in the lower compartment of the transwell plate, and the TEER (upper graph) was measured at the indicated times. **K** The culture supernatants from either mock-infected or JEV-infected astrocytes were collected and mixed with an equal volume of fresh medium (Mock-CM/JEV-CM) and added to confluent hBMECs in the upper chamber of the Transwell plate. Then, the TEER was measured at the indicated times. **L**, **M** PRR-knockdown hBMECs were seeded onto the upper chamber of the Transwell plate until confluent. The TEER (upper graph) was measured without treatment (**L**) or with mock-CM/JEV-CM treatment (**M**) at the indicated times. Data are represented as the mean values ± SEMs from three independent experiments. *p < 0.05; **p < 0.01; ****p < 0.0001; ns, not significant

The detection of viral RNA by PRRs such as TLRs and RIG-I-like receptors (RLRs) is well documented, and TLR3, RIG-I, and MDA5 are widely used and are enriched in our RNA-seq data. Consistently, the enrichment was confirmed at the mRNA and protein levels in the hBMECs responding to JEV infection (Fig. 4A–C). To further investigate the role of PRRs in hBMECs, stable cell lines with short hairpin RNA (shRNA)-mediated knockdown of TLR3 (shTLR3), RIG-I (shRIG-I), and MDA5 (shMDA5) were generated. As expected, the expression of each PRR (TLR3, RIG-I, and MDA5) was efficiently knocked down in the corresponding cell lines (Fig. 4D, E). Then, the CCK-8 assay revealed undescended cell viability in each PRR-knockdown hBMEC compared to shCTL hBMECs (Fig. 4F). As the initiator in the host innate immune pathway, the ability of PRRs to modulate IFIT expression was next investigated. As shown, in each PRR-knockdown hBMEC, IFIT1, IFIT2, and IFIT3 were lowered in expression during JEV infection at both the transcription and translation levels, whereas inhibition of IFIT5 only appeared in shTLR3 hBMECs (Fig. 4G, H). Taken together, the activation of PRRs in hBMECs is involved in IFIT induction during JEV infection.

Host innate immune factors diversely modulate BBB permeability [21, 50]. Since PRRs initiate innate immune signals, we next investigated their role in modulating BBB integrity via the in vitro transwell BBB model. Quantitative measurements of TEER (Fig. 4I–L) were performed in hBMEC monoculture, cocultured hBMECs with astrocytes (U251), and PRR-knockdown hBMECs. As displayed, direct infection of JEV in hBMECs did not alter the hBMEC barrier integrity (Fig. 4I). In contrast, a compromised endothelial barrier appeared in both hBMECs cocultured with JEV-infected astrocytes (Fig. 4J) and hBMEC monocultures treated with JEV-CM (Fig. 4K). In PRR-knockdown hBMECs, without additional treatment, no significant alteration in TEER was observed compared to shCTL hBMECs (Fig. 4L). Upon treatment with JEV-CM, the knockdown of TLR3 resulted in a slower decrease in TEER. In contrast, JEV-CM caused an accelerated breakdown of hBMEC barrier integrity in shRIG-I and shMDA5 hBMECs (Fig. 4M). Altogether, the obtained results indicated that the activation of PRRs in hBMECs exerts the function of both inducing IFIT expression and differentially regulating hBMEC barrier integrity.

### JEV infection-activated IRF is downstream of PRRs, which may account for the IFN-dependent induction of IFITs

Members of the IFN-regulatory factor (IRF) family regulate gene expression and are pivotal to IFN immune responses. RNA-seq data of JEV-infected hBMECs demonstrated the enrichment of IRF1 and IRF7, which was further confirmed by RT-qPCR results (Additional file 6: Fig. S6A). Based on the critical role in IFN signaling [51, 52], the expression of IRF3 was also determined. At the protein level, JEV infection induced the accumulation of IRF7, IRF1, and phosphorylated IRF3 (Additional file 6: Fig. S6B). To investigate whether the altered IRF family members were correlated with viral-activated PRRs, shTLR3, shRIG-I, and shMDA5 hBMECs were employed. As demonstrated, the protein expression of IRF7, IRF1, and phosphorylated IRF3 significantly declined in each PRR-knockdown hBMEC compared to shCTL hBMEC (Additional file 6: Fig. S6C). Together, it can be speculated that IRF family members account for the induction of IFN-dependent IFITs.

### Identification of type I/type III interferons in maintaining hBMEC barrier integrity and the induction of IFITs

**Fig. 5:**
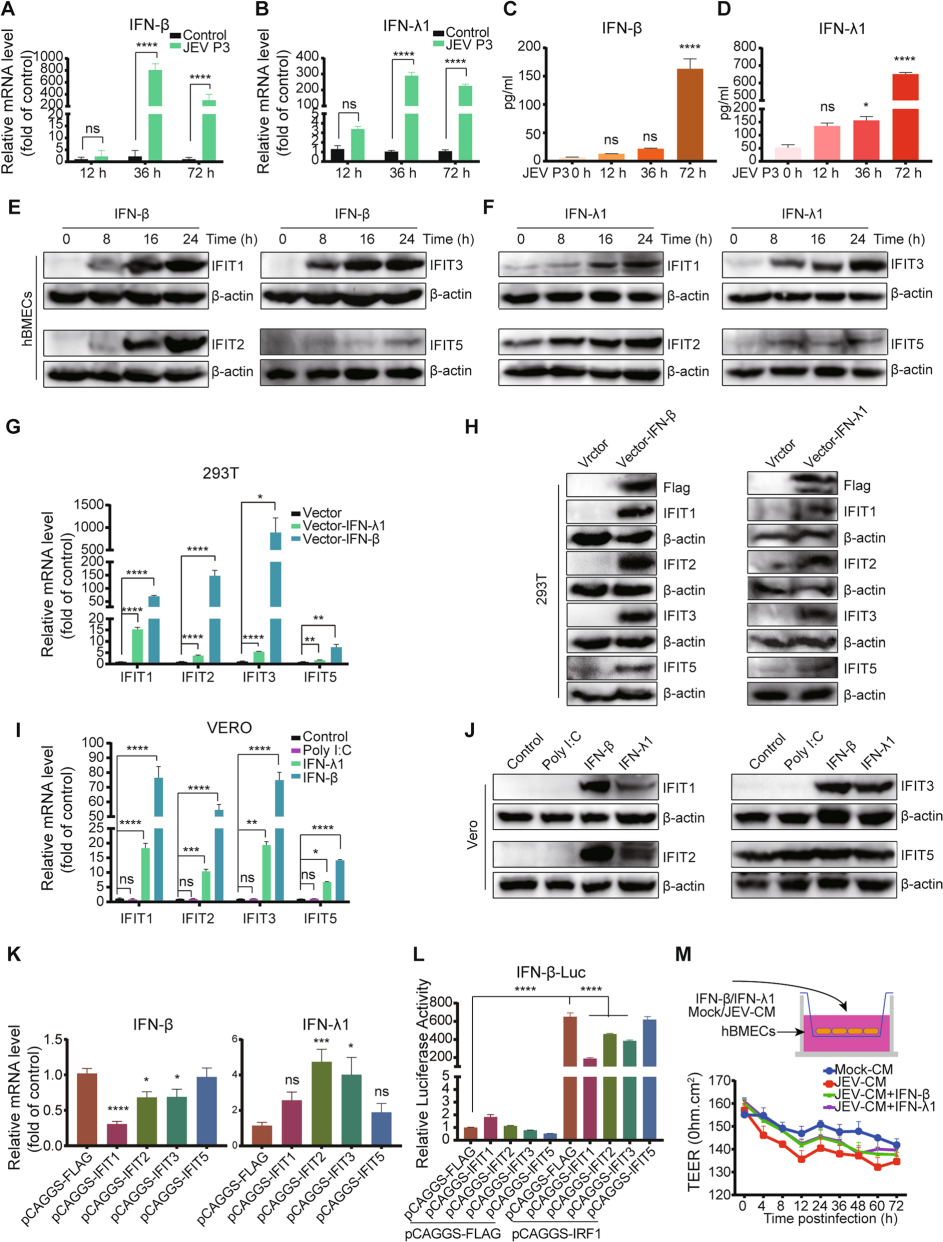
The role of IFNs in IFIT induction and hBMEC barrier maintenance. hBMECs were infected with JEV P3, the cells/cell culture supernatants were harvested at 0, 12, 36, and 72 hpi, and the expression of IFN-β/IFN-λ1 at the mRNA and protein levels was determined by utilizing RT-qPCR (**A**, **B**) and ELISA (**C**, **D**). **E**, **F** hBMECs were treated with 10 ng/ml rhIFN-β (**E**) or 100 ng/ml rhIFN-λ1 protein (**F**) for different time points, and IFIT expression was measured by Western blotting. **G**, **H** HEK-293T cells were transfected with either empty vector plasmid (vector) or IFN-β (Vector-IFN-β)/IFN-λ1 (Vector-IFN-λ1) expression plasmid as indicated, and IFIT expression was measured by performing RT-qPCR (**G**) and Western blotting (**H**). **I**, **J** Vero cells were treated with poly I: C or rhIFN-β/rhIFN-λ1 protein as indicated, and IFIT expression was measured by performing RT-qPCR (**I**) and Western blotting (**J**). **K** HEK-293T cells were transfected with either empty vector plasmid (vector) or plasmids expressing IFIT1 (Vector-IFIT1), IFIT2 (Vector-IFIT2), IFIT3 (Vector-IFIT3), and IFIT5 (Vector-IFIT5) as indicated, and IFN-β and IFN-λ1 mRNA expression was measured by performing RT-qPCR. (L) Dual-luciferase reporter assays detected IRF1 binding to the IFN-β promotor in IFIT-overexpressing HEK-293T cells. HEK-293T cells were transfected with vector-IFIT1, vector-IFIT2, vector-IFIT3, vector-IFIT5, or empty vector plasmid to generate overexpressed cell lines. Simultaneously, empty vector plasmid or vector-IRF1 and IFN-β-Luc alone with pRL-TK plasmids were cotransfected into corresponding overexpressed cell lines. **M** hBMECs were cultured in the upper chamber of the transwell plate and pretreated with rhIFN-β and rhIFN-λ1 protein, followed by treatment with mock-CM or JEV-CM, and then the TEER (upper graph) was determined at the indicated times. Data are represented as the mean values ± SEMs from three independent experiments. *p < 0.05; **p < 0.01; ***p < 0.001; ****p < 0.0001; ns: not significant

Previous reports revealed that the induction of IFITs occurs in both an IFN-dependent and IFN-independent manner [53, 54]. Based on our RNA-seq data and RT-qPCR analysis, accumulation of type I and type III interferons (IFN-β and IFN-λ1) occurred in hBMECs under JEV infection (Fig. 5A, B), accompanied by consistent upregulation at the protein expression level (Fig. 5C, D). Then, it was questioned whether the induction of IFITs in hBMECs relies on IFN signaling. The cells were pretreated with rhIFN-β and rhIFN-λ1, and a dose-dependent upregulation of IFITs mRNA was observed (Additional file 6: Fig. S7A, C). Notably, although IFIT mRNA expression reached a maximum at 8 h post-JEV infection (Additional file 6: Fig. S7B, D), its protein expression was lower at 8 hpi than at subsequent time points, indicating the necessity of prolonged stimulation with rhIFN-β and rhIFN-λ1 for maintaining IFIT expression (Fig. 5E, F). To explore whether IFN-dependent expression of IFITs ubiquitously exists in cells, HEK-293T cells and Vero cells were employed since there is no production of IFNs in Vero cells upon viral infection, but they can respond to exogenous IFNs [55, 56]. As depicted in Fig. 5G and H, overexpression of both IFN-β and IFN-λ1 in HEK-293T cells immensely enhanced IFIT expression at the mRNA and protein levels (Fig. 5G, H). Meanwhile, the emergence of upregulated IFITs was observed in IFN-β- and IFN-λ1- but not poly I: C-treated Vero cells (Fig. 5I, J), which further confirmed that IFN signaling is responsible for the induction of IFITs.

Since IFIT1 could negatively regulate IFN expression [57], the interrelationship between other IFITs and IFNs was further investigated. As the RT-qPCR results showed, the overexpression of IFIT1, IFIT2, and IFIT3 restricted IFN-β but not IFN-λ1 induction in HEK-293 T cells (Fig. 5K). The luciferase assay results further illustrated that IFIT1, IFIT2, and IFIT3 inhibited IFN-β promoter activity (Fig. 5L). These observations demonstrated that IFIT1, IFIT2, and IFIT3 negatively modulate IFN-β expression.

It was reported that IFNs are involved in BBB regulation among host innate immune factors [14, 22]. An in vitro transwell BBB model was employed to determine whether the enriched type I/type III IFNs could contribute to the undamaged barrier integrity of hBMECs upon JEV infection. rhIFN-β/rhIFN-λ1 was added to the upper part of the chamber prior to treatment with JEV-CM in hBMECs, which mitigated the barrier disruption induced by JEV-CM (Fig. 5M), further demonstrating the critical role of IFNs in barrier maintenance. All these data supported the participation of IFNs (IFN-β and IFN-λ1) in both hBMEC barrier maintenance and IFIT induction.

### JAK/STAT pathway was involved in the IFN-dependent induction of IFITs

**Fig. 6:**
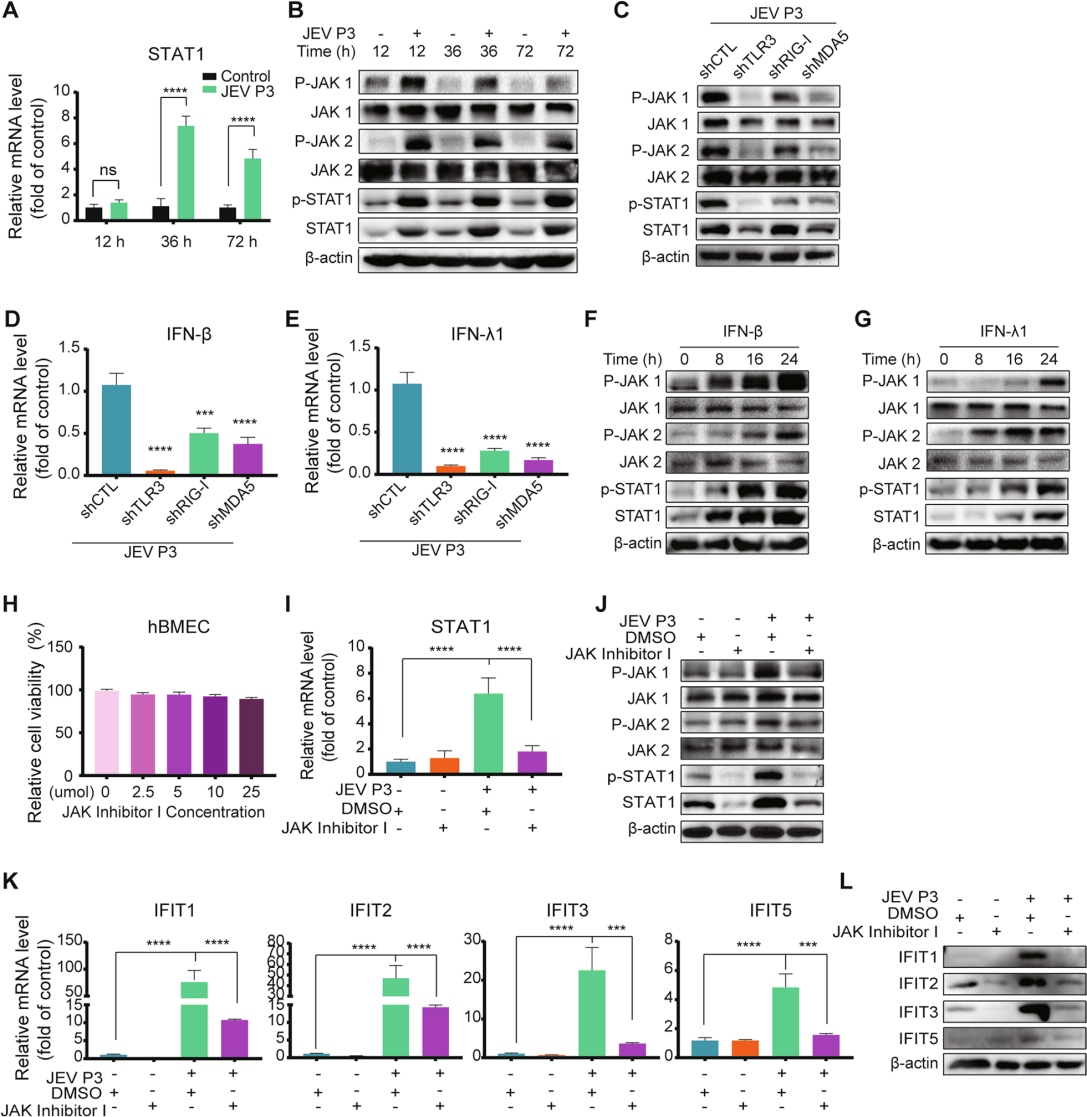
The role of the JAK/STAT pathway in IFIT induction. **A**, **B** hBMECs were infected with JEV P3 for different time points, and the mRNA of STAT1 (**A**) and the protein of JAK1/p-JAK1, JAK2/p-JAK2, and STAT1/p-STAT1 (**B**) were measured. **C** The protein expression of JAK1/p-JAK1, JAK2/p-JAK2, and STAT1/p-STAT1 was measured in PRR-knockdown hBMECs under JEV infection. **D**, **E** IFN-β and IFN-λ1 mRNA levels were measured in PRR-knockdown hBMECs under JEV infection. **F**, **G** hBMECs were treated with rhIFN-β or rhIFN-λ1 protein for different time points, and JAK1/p-JAK1, JAK2/p-JAK2, and STAT1/p-STAT1 protein expression was measured. **H** hBMECs were treated with the carrier control DMSO or JAK inhibitor I at various concentrations for 72 h, and cell cytotoxicity was analyzed. **I**, **J** hBMECs were pretreated with carrier control DMSO or 5 μM JAK inhibitor I, followed by JEV P3 infection, and the mRNA of STAT1 (**I**) and the protein of JAK1/p-JAK1, JAK2/p-JAK2, and STAT1/p-STAT1 (**J**) were determined. **K**, **L** hBMECs were pretreated with the carrier control DMSO or 5 μM JAK inhibitor I, followed by JEV P3 infection, and IFIT mRNA (**K**) and protein (**L**) expression was measured. Data are represented as the mean values ± SEMs from three independent experiments. ***p < 0.001; ****p < 0.0001; ns: not significant

PRR (RIG-I)-STAT1 signaling has been previously reported in brain organoid antiviral immunity in JEV infection [58]. RNA-seq and RT-qPCR data suggested that JEV stimulated STAT1 transcription (Additional file 5: Table S6, Fig. 6A). JEV infection induced the phosphorylation of JAK1, JAK2, and STAT1 in hBMECs, indicating activation of the JAK/STAT pathway (Fig. 6B). Furthermore, the activation of the JAK/STAT pathway and IFN-β/IFN-λ1 induction were weakened in PRR-knockdown hBMECs under JEV infection (Fig. 6C–E). Now, the question arises of how IFNs regulate the PRR-mediated JAK/STAT pathway. After treatment with rhIFN-β and rhIFN-λ1, the phosphorylation of JAK1, JAK2, and STAT1 was upregulated, indicating that the JAK/STAT pathway is downstream of IFNs (Fig. 6F, G). Then, a specific inhibitor, JAK inhibitor I, was employed to investigate the role of the JAK/STAT pathway in IFIT induction. The CCK-8 assay and the inhibition effects identified 5 μM as the practical working concentration (Fig. 6H, I, J), which significantly impaired the expression of JEV-induced IFITs (Fig. 6K, L). These results strongly demonstrated the involvement of the JAK/STAT pathway in IFN-dependent IFIT induction.

### IFITs are not responsible for maintaining hBMEC barrier integrity

**Fig. 7:**
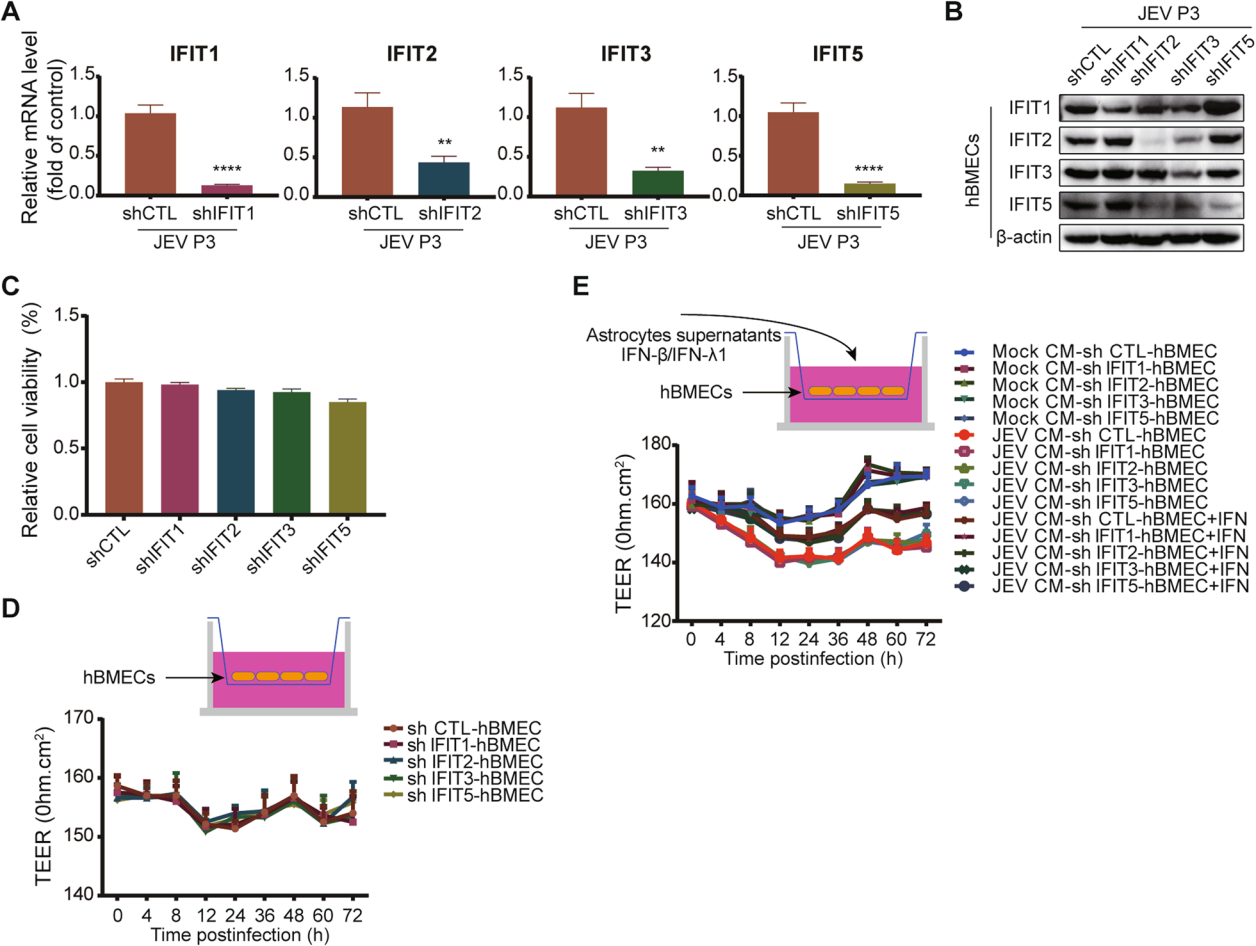
IFITs are nonessential in hBMEC barrier integrity. **A**, **B** The expression of IFITs was assessed by RT-qPCR (A) and Western blotting (**B**) in IFIT-knockdown hBMECs under JEV infection. **C** After serum-free starvation, cell viability was determined in IFIT-knockdown hBMECs. **D**, **E** IFIT-knockdown hBMECs were seeded onto the upper chamber of the Transwell plate until confluent. At the indicated times, the TEER (upper graph) was measured without treatment (**D**) or with rhIFN-β and rhIFN-λ1 protein treatment prior to the addition of JEV-CM (**E**). Data are represented as the mean values ± SEMs from three independent experiments. **p < 0.01; ****p < 0.0001

The results mentioned above have revealed a PRR-IFN-dependent induction of IFITs. Considering the effect of PRR-IFNs on hBMEC barrier integrity and the complex interrelationship between IFNs and IFITs, we next determined the role of IFITs in modulating the barrier function of hBMECs. Stable cell lines with shRNA-mediated knockdown of IFIT1 (shIFIT1), IFIT2 (shIFIT2), IFIT3 (shIFIT3), and IFIT5 (shIFIT5) were generated in hBMECs. The knockdown efficacy was confirmed at the mRNA and protein levels (Fig. 7A, B). More than 90% of cell viability was maintained under the knockdown of IFITs in hBMECs (Fig. 7C). Then, the TEER in IFIT-knockdown hBMECs was measured using the in vitro transwell BBB model. As illustrated, no noticeable difference appeared between shCTL hBMECs and IFIT-knockdown hBMECs without additional treatment (Fig. 7D). However, the decreased TEER was observed in IFIT-knockdown hBMECs caused by JEV-CM, which could be partially rescued by treatment with rhIFN-β and rhIFN-λ1 (Fig. 7E), indicating a nonessential role of IFITs in the maintenance of hBMEC barrier integrity by IFNs.

## Discussion

**Fig. 8:**
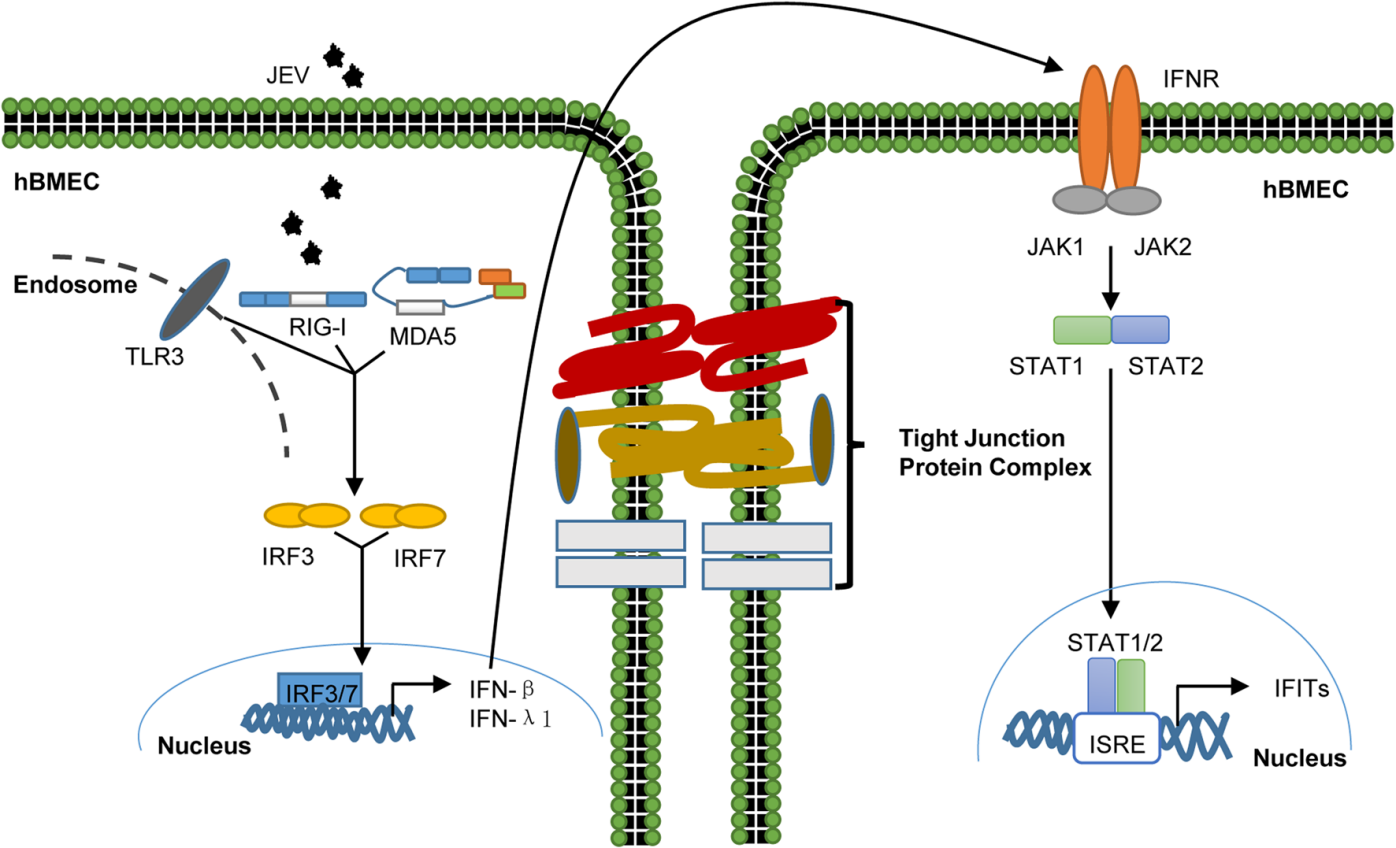
Schematic presentation of host innate immune factor-mediated intracellular communication for inducing IFIT expression, restricting JEV infection, and modulating hBMEC barrier integrity. During infection, hBMECs employed PRRs (TLR3, RIG-I, and MDA5) to sense JEV and subsequently triggered IRF phosphorylation and translocation to the nucleus, leading to the synthesis and secretion of IFNs, which induced IFIT expression in a JAK/STAT-dependent manner. Additionally, these upregulated immune factors differentially regulate hBMEC barrier integrity

Growing evidence suggests that permeabilizing and spreading from the endothelium is a critical initial step for neuroviruses to bypass the BBB and cause CNS inflammation [8, 9, 12, 59, 60]. However, to date, only a few reports are available on mapping the comprehensive gene expression profiles of BBB endothelial cells responding to Flaviviridae, especially JEV. For the first time, RNA-seq was performed in JEV-infected hBMECs, which provided more in-depth information on overall gene expression and triggered cell signaling. As presented in Fig. 8, hBMECs first employed PRRs (TLR3, RIG-I, and MDA5) to sense JEV and then initiated the IRF/IFN response to induce IFIT expression in a JAK/STAT-dependent manner. In addition to the well-characterized function of sensing viruses, the knockdown of distinct PRRs in hBMECs exerts different regulatory effects on endothelial barrier integrity. Downstream of PRRs, IFNs and IFITs exhibited antiviral activity in JEV infection. Notably, in an in vitro BBB model, treatment with rhIFN-β and rhIFN-λ1 mitigated the JEV-CM-compromised hBMEC barrier, indicating the protective role of IFNs, which aligns with previous studies [14, 24, 61, 62]. However, IFITs were dispensable in maintaining the hBMEC barrier, despite their complex interrelationship with IFNs.

Similar to the previous result [9], in our in vitro BBB model, direct infection of JEV failed to destroy the hBMEC barrier, while the basolateral release of infectious particles from infected BMECs may be sufficient for virus invasion without a remarkable CPE [63]. These findings suggested a way for JEV to permeabilize the BBB and spread into the CNS from BMECs. Upon natural infection in humans, few studies have confirmed the infection of BMECs owing to technical issues in the analysis of brain tissues [64, 65]. These events highlight the necessity of exploring the infection of human BMECs in vitro.

In the BBB, BMECs are in the first line of contact with JEV or JEV-infected cells. The innate immune responses are immediately initiated in host cells upon viral infection, including hBMECs [33, 48]. Although hBMECs are widely accepted as a constituent of the BBB, immune activation generally occurs with stimulation [66]. The RNA-seq data in this study provided information on the activated hBMEC immune response in defending against JEV invasion, including the JAK/STAT signaling pathway, cytokine‒cytokine receptor interaction, Toll-like receptor signaling pathway, RIG-I-like receptor signaling pathway, etc. Consistent with previous reports, PRRs (TLRs and RLRs) positively regulate IRF/IFN signaling to restrict JEV infection [67] and induce the expression of IFITs [68]. RLR signaling, including RIG-I and MDA5, has been implicated in viral RNA recognition, including that of JEV [69]. Then, IRF (IRF1, IRF3, and IRF7) signaling was activated and bound to response elements stimulated by IFN and located on the promoter of IFN genes [70] to induce ISG (ISG15, HERC5, IFITM1, BST2, OAS1, OAS2, OAS3, OASL, GBP1, GBP4, IFI44, TRIM22, IFI35, IFIT1, IFIT2, IFIT3, and IFIT5) expression in the hBMEC response to JEV infection. ISGs take on diverse roles in host cells against viral infection [71]. The antiviral activity of ISG15 is well characterized in resistant Flaviviruses, including JEV, Dengue fever virus, and West Nile virus (WNV) [72, 73]. The Mx GTPase pathway exerts an antiviral effect against various viruses [74–76], but is nonessential for IFN-α against JEV [77]. Although IFI35 was identified as a contributor to other Flaviviridae infections [46], the overexpression of IFI35 failed to boost JEV infection in this study, which might be due to the pleiotropic functions of genes in different viral infections and distinct cell types.

Enriched IFITs caught our attention with their multiple functions in participating in brain cells’ antiviral immune response and viral encephalitis [18, 78–81]. Consistent with the results in human glomerular endothelial cells, IFITs were found to be strongly induced in poly I: C treatment and viral infection in previous reports [35, 82] and this study. In addition, they can be induced by both IFN-dependent and IFN-independent mechanisms [53, 67]. By overexpressing IFNs in HEK-293T cells or treating hBMECs/Vero cells with rhIFN-β/rhIFN-λ1 protein, we observed the upregulation of IFITs, which is in line with the essential role of IFNs in regulating IFIT expression [83]. IFITs exert antiviral functions in several viral infections, including severe acute respiratory syndrome coronavirus 2 (SARS-CoV-2), human cytomegalovirus (HCMV), and WNV [19, 84–86]. IFIT1 has been found to directly recognize 5′-triphosphate viral RNA to restrict JEV infection, as host mRNAs lack this feature [28]. Similar inhibitory effects also appeared for other IFITs in this research, including IFIT2, IFIT3, and IFIT5. In contrast, NF-κB-dependent IFIT3 was found to prompt hepatitis B virus (HBV) infection [87]. Human and murine IFIT1 failed to restrict negative-sense RNA viral replication [88]. Intriguingly, in our study, overexpression of IFITs (IFIT1/IFIT3) inhibited IFN-β activation, in accordance with previously reported results [57, 89], indicating complex crosstalk between IFITs and IFNs.

BBB disruption occurs in the JEV-infected mouse model and in the in vitro BMECs-astrocytes coculture BBB model [29, 90]. The supernatants of JEV-infected astrocytes disrupt endothelial barrier integrity in vitro via inflammatory cytokines [30], which was observed in our study. As a highly specialized structure of the BBB, PRR expression not only senses viral PAMPs, but also modulates BBB homeostasis [50]. In line with a previous report [21], the knockdown of PRRs (RIG-I and MDA5) accelerated the disruption of hBMEC barrier integrity caused by JEV-CM in an in vitro BBB model. On the basis of our findings and the general trends observed in the literature, activated PRRs (RIG-I and MDA5) are sufficient to maintain the BMEC barrier function dominated by induced IFNs. Treatment with distinct PRR agonists leads to varied consequences on the BMEC barrier [21], indicating a complicated correlation between the BBB and distinct PRRs. Consistent with our data, activation of TLR3 was observed to boost barrier dysregulation in BMECs [21], indicating a disruptive role of TLR3 in BBB maintenance. However, the specific role of TLR3 in BBB regulation remains controversial, as another report failed to link TLR3 activation to BBB disruption [91]. These inconsistencies may be due to the multifunctional genes, distinct viral infections, infection conditions, and various downstream signals of PRRs [92].

IFNs are predominant in regulating the BBB, which can be induced via activated PRRs in hBMECs responding to JEV infection. Treatment with IFN-β protects the BBB in relapsing–remitting multiple sclerosis (RRMS) patients [93]. IFN-β dose-dependently stabilized endothelial barrier integrity with increased TEER in an in vitro BBB model of brain capillary endothelial cells and rat astrocytes on the reversed side [25]. Functionally, the protective effect of IFN-β can maintain endothelial cell barrier integrity as well as counteract the impact of barrier-disrupting factors (such as inflammatory cytokines TNF-α and IL-1β) [21, 50], which was in line with our result in the in vitro transwell model. Type III IFN (IFN-λ) exerts a protective effect by stabilizing the BBB and limiting virus neuroinvasion [26]. Consistently, treatment with rhIFN-λ in hBMECs decreased JEV-CM-induced hBMEC barrier damage in this study. Considering the sophisticated interrelation between IFNs and IFITs, the question arises as to whether IFITs are involved in endothelial barrier regulation. As in prior studies, an accumulation of IFIT2 has been found in JE [94, 95], and IFIT2 deficiency triggered uncontrolled neurotropic coronavirus infection with enhanced encephalitis [81]. However, treatment with IFNs in IFIT-knockdown hBMECs still subsided JEV-CM-induced endothelial barrier disruption, suggesting a nonessential role of IFITs in maintaining the barrier integrity of hBMECs. Although IFN’s maintenance of the BBB was validated in our and others’ models, the specific mechanism and function of downstream ISGs in IFN-mediated BBB maintenance have yet to be determined and require further study. Currently, strand-specific sequencing of RNA (ssRNA-seq) has emerged as a powerful tool in profiling complex transcriptomes [96]. Additionally, single-cell RNA-seq (scRNA-seq) analysis has been employed in studying the BBB in vivo [97]. Therefore, it is essential to utilize novel technologies to delve deeper into the specific role of IFN in regulating BBB integrity in vivo and to elucidate whether it could serve as a potential therapeutic target during JE.

## Conclusions

In summary, this research provided the first comprehensive analysis of host‒pathogen interactions in hBMECs responding to JEV infection by RNA-seq. We identified the innate immune pathway PRR-IRF/IFN-JAK/STAT-IFIT signaling cascade in BMECs effectively against JEV infection. Additionally, different PRRs differentially regulate the endothelial barrier, and IFNs protect the hBMEC barrier from JEV-CM (including inflammatory cytokines), whereas IFITs are not essential in IFN-mediated endothelial barrier maintenance. Collectively, understanding the role of type I/type III IFN and related factors in JEV infection and BBB integrity may help develop a new therapeutic strategy for CNS inflammatory responses in JE and other neuroinvasive diseases in the future.

### Supplementary Information

**Additional file 1: Table S1.** Primers employed for RT-qPCR in this study. **Table S2.** shRNA oligonucleotide sequences in this study.

**Additional file 2: Fig. S1.** Principal component analysis (PCA) of JEV-infected hBMECs at 12, 36, and 72 hpi. (A) Note: PC1 illustrates the differences among JEV-infected samples; PC2 shows differences between mock-infected and JEV-infected samples at different time points, including 12, 36, and 72 hpi.

**Additional file 3: Table S3.** RNA-seq quantified in JEV-infected hBMECs at 12, 36, and 72 hpi.

**Additional file 4: Fig. S2.** Volcano plot of global DEGs at different time points. V P3 vs. Mock 12 h (A), JEV P3 vs. Mock 36 h (B), JEV P3 vs. Mock 72 h (C). Purple dots (right) and red dots (left) represent significantly upregulated and downregulated genes, respectively. Blue dots (middle) represent insignificantly expressed genes. **Fig. S3.** GO and KEGG enrichment statistics. The top 30 GO terms were clustered into 3 categories, including cellular component (CC), molecular function (MF), and biological process (BP), mock VS JEV 12 h (A), mock VS JEV 36 h (C), and mock VS JEV 72 h (E). Top 20 Kyoto Encyclopedia of Genes and Genomes (KEGG) pathways in JEV-infected hBMECs, mock vs JEV 12 h (B), mock vs JEV 36 h (D), and mock vs JEV 72 h (F).

**Additional file 5: Table S4.** Information on the GO pathways of the DEGs in JEV-infected hBMECs at 12, 36, and 72 hpi. **Table S5.** Information on the KEGG pathways of the DEGs in JEV-infected hBMECs at 12, 36, and 72 hpi. **Table S6.** Global transcriptional response of JEV-infected hBMECs.

**Additional file 6: Fig. S4.** JEV infection induced the upregulation of IFI35 in hBMECs. hBMECs were infected with JEV P3 for the indicated time, and the expression of IFI35 at the mRNA level (A) and protein level (B) was measured by performing RT-qPCR and Western blotting, respectively. (C) HEK293T cells were transfected with an empty vector (vector) or IFI35 (Vector-IFI35) as indicated, and the expression of IFI35 and JEV-E at the protein level was measured by Western blotting. Data are represented as the mean values ± SEMs from three independent experiments. **p < 0.01; ****p < 0.0001. **Fig. S5.** JEV infection triggers the induction of ISGs in hBMECs, Related to Table 2. The hBMECs were infected with JEV P3 for the indicated time, and mRNA levels of IFN-λ2,3, IFITM1, ISG15, OAS1, OAS2, OASL, GBP4, CXCL10, USP18, IFI44, and CCL5 in these cells were quantified by RT-qPCR (A), and the protein levels of USP18, IFITM1, and ISG15 were measured by Western blotting (B). **Fig. S6.** JEV infection induced IRF activation in hBMECs. The hBMECs were infected with JEV P3 for the indicated time, and the expression of IRF7 and IRF1 at the mRNA level (A) and IRF7, IRF1, and IRF3/p-IRF3 at the protein level (B) were measured by performing RT-qPCR and Western blotting, respectively. (C) Cells were infected with JEV P3 for 36 h, and the protein levels of IRF7, IRF1, and IRF3/p-IRF3 were measured in shCTL, shTLR3, shRIG-I, and shMDA5 hBMECs, representing significantly upregulated and downregulated genes, respectively. Blue dots (middle) represent insignificantly expressed genes. Data are represented as the mean values ± SEMs from three independent experiments. ** p < 0.01; ****, p < 0.0001. **Fig. S7.** IFN-β/IFN-λ1 treatment induced IFIT mRNA expression in hBMECs. (A, C) hBMECs were treated with distinct concentrations of rhIFN-β/rhIFN-λ1 protein, and IFIT mRNA expression was measured. (B, D) Cells were treated with 10 ng/ml rhIFN-β (B) or 100 ng/ml rhIFN-λ1 protein (D) for different time points, and IFIT expression was measured by performing RT-qPCR. Data are represented as the mean values ± SEMs from three independent experiments. **p < 0.01; ****p < 0.0001.

**Additional file 7: Table S7.** List of antibodies used in this study.

## Data Availability

All datasets generated and/or analyzed during this study are available from the corresponding author upon reasonable request. The RNA-seq data in this study have been deposited in the NCBI SRA database under accession number PRJNA936274.

## Abbreviations

JEV: Japanese encephalitis virus
BBB: Blood‒brain barrier
hBMECs: Human brain microvascular endothelial cells
CNS: Central nervous system
RNA-Seq: RNA-sequencing
RT-qPCR: Quantitative reverse transcription-polymerase chain reaction
DEGs: Differentially expressed genes
GO: Gene Ontology
KEGG: Kyoto Encyclopedia of Genes and Genomes
DMEM: Dulbecco’s modified Eagle’s medium
FBS: Fetal bovine serum
IFN: Interferon
MOI: Multiplicity of infection
IF: Immunofluorescence
Hpi: Hours post-infection
ISGs: Interferon-stimulated genes
Mock-CM: Mock-infected astrocyte supernatants mixed with an equal volume of fresh medium
JEV-CM: JEV-infected astrocyte supernatants mixed with an equal volume of fresh medium

## References

[1] Ghosh D, Basu A (2009). Japanese encephalitis-a pathological and clinical perspective. PLoS Negl Trop Dis.

[2] Wang H, Liang G (2015). Epidemiology of Japanese encephalitis: past, present, and future prospects. Ther Clin Risk Manag.

[3] Kaur R, Vrati S (2003). Development of a recombinant vaccine against Japanese encephalitis. J Neurovirol.

[4] Deberardinis RJ, Sayed N, Ditsworth D, Thompson CB (2008). Brick by brick: metabolism and tumor cell growth. Curr Opin Genet Dev.

[5] Eigenmann DE, Xue G, Kim KS, Moses AV, Hamburger M, Oufir M (2013). Comparative study of four immortalized human brain capillary endothelial cell lines, hCMEC/D3, hBMEC, TY10, and BB19, and optimization of culture conditions, for an in vitro blood-brain barrier model for drug permeability studies. Fluids Barriers CNS.

[6] Goasdoué K, Miller SM, Colditz PB, Björkman ST (2017). Review: The blood-brain barrier; protecting the developing fetal brain. Placenta.

[7] Stebbins MJ, Gastfriend BD, Canfield SG (2019). Human pluripotent stem cell-derived brain pericyte-like cells induce blood-brain barrier properties. Sci Adv.

[8] Al-Obaidi MMJ, Bahadoran A, Har LS, Mui WS, Rajarajeswaran J, Zandi K, Manikam R, Sekaran SD (2017). Japanese encephalitis virus disrupts blood-brain barrier and modulates apoptosis proteins in THBMEC cells. Virus Res.

[9] Lai CY, Ou YC, Chang CY, Pan HC, Chang CJ, Liao SL, Su HL, Chen CJ (2012). Endothelial Japanese encephalitis virus infection enhances migration and adhesion of leukocytes to brain microvascular endothelia via MEK-dependent expression of ICAM1 and the CINC and RANTES chemokines. J Neurochem.

[10] Filgueira L, Lannes N (2019). Review of Emerging Japanese Encephalitis Virus: New Aspects and Concepts about Entry into the Brain and Inter-Cellular Spreading. Pathogens.

[11] Li J, Wang Y, Wang X, Ye L, Zhou Y, Persidsky Y, Ho W (2013). Immune activation of human brain microvascular endothelial cells inhibits HIV replication in macrophages. Blood.

[12] Mladinich MC, Schwedes J, Mackow ER (2017). Zika Virus Persistently Infects and Is Basolaterally Released from Primary Human Brain Microvascular Endothelial Cells. MBio.

[13] Higazy D, Lin X, Xie T, Wang K, Gao X, Cui M (2022). Altered gene expression in human brain microvascular endothelial cells in response to the infection of influenza H1N1 virus. Anim Dis..

[14] Lazear HM, Daniels BP, Pinto AK, Huang AC, Vick SC, Doyle SE, Gale M, Klein RS, Diamond MS (2015). Interferon-λ restricts West Nile virus neuroinvasion by tightening the blood-brain barrier. Sci Transl Med.

[15] Akira S, Uematsu S, Takeuchi O (2006). Pathogen recognition and innate immunity. Cell.

[16] Stark GR, Darnell JE (2012). The JAK-STAT pathway at twenty. Immunity.

[17] Cheng Y, Medina A, Yao Z, Basu M, Natekar JP, Lang J, Sanchez E, Nkembo MB, Xu C, Qian X (2022). Intrinsic antiviral immunity of barrier cells revealed by an iPSC-derived blood-brain barrier cellular model. Cell Rep.

[18] Fensterl V, Wetzel JL, Ramachandran S, Ogino T, Stohlman SA, Bergmann CC, Diamond MS, Virgin HW, Sen GC (2012). Interferon-induced Ifit2/ISG54 protects mice from lethal VSV neuropathogenesis. PLoS Pathog.

[19] Pichlmair A, Lassnig C, Eberle CA, Górna MW, Baumann CL, Burkard TR, Bürckstümmer T, Stefanovic A, Krieger S, Bennett KL (2011). IFIT1 is an antiviral protein that recognizes 5'-triphosphate RNA. Nat Immunol.

[20] Tan XF, Chen Q, Hua SH, Yip GW (2021). Roles of Interferon Induced Protein with Tetratricopeptide Repeats (IFIT) Family in Cancer. Curr Med Chem.

[21] Daniels BP, Holman DW, Cruz-Orengo L, Jujjavarapu H, Durrant DM, Klein RS (2014). Viral pathogen-associated molecular patterns regulate blood-brain barrier integrity via competing innate cytokine signals. MBio.

[22] Kraus J, Oschmann P (2006). The impact of interferon-beta treatment on the blood-brain barrier. Drug Discov Today.

[23] Wilkins C, Woodward J, Lau DT, Barnes A, Joyce M, McFarlane N, McKeating JA, Tyrrell DL, Gale M (2013). IFITM1 is a tight junction protein that inhibits hepatitis C virus entry. Hepatology.

[24] Kraus J, Ling AK, Hamm S, Voigt K, Oschmann P, Engelhardt B (2004). Interferon-beta stabilizes barrier characteristics of brain endothelial cells in vitro. Ann Neurol.

[25] Gaillard PJ, van Der Meide PH, de Boer AG, Breimer DD (2001). Glucocorticoid and type 1 interferon interactions at the blood-brain barrier: relevance for drug therapies for multiple sclerosis. NeuroReport.

[26] Wells AI, Coyne CB (2018). Type III interferons in antiviral defenses at barrier surfaces. Trends Immunol.

[27] Fu S, Yu M, Xu H, Liu Q, Li X, Wang Y, Chen Y, Meng L, Qiu Y, Jing X (2021). Genome-wide transcription analysis of electroacupuncture precondition-induced ischemic tolerance on SD rat with ischemia-reperfusion injury. Front Genet.

[28] Kimura T, Katoh H, Kayama H, Saiga H, Okuyama M, Okamoto T, Umemoto E, Matsuura Y, Yamamoto M, Takeda K (2013). Ifit1 inhibits Japanese encephalitis virus replication through binding to 5' capped 2'-O unmethylated RNA. J Virol.

[29] Li F, Wang Y, Yu L, Cao S, Wang K, Yuan J, Wang C, Wang K, Cui M, Fu ZF (2015). Viral infection of the central nervous system and neuroinflammation precede blood-brain barrier disruption during Japanese encephalitis virus infection. J Virol.

[30] Chang CY, Li JR, Chen WY, Ou YC, Lai CY, Hu YH, Wu CC, Chang CJ, Chen CJ (2015). Disruption of in vitro endothelial barrier integrity by Japanese encephalitis virus-Infected astrocytes. Glia.

[31] Wang X, Maruvada R, Morris AJ, Liu JO, Wolfgang MJ (2016). Sphingosine 1-phosphate activation of EGFR as a novel target for meningitic *Escherichia coli* penetration of the blood-brain barrier. PLos Pathogen.

[32] Yang B, Yin P, Yang R, Xu B, Fu J, Zhi S, Dai M (2020). Holistic insights into meningitic *Escherichia coli* infection of astrocytes based on whole transcriptome profiling. Epigenimics.

[33] Zhang YG, Chen HW, Zhang HX, Wang K, Su J, Chen YR, Wang XR, Fu ZF, Cui M (2022). EGFR activation impairs antiviral activity of interferon signaling in brain microvascular endothelial cells during Japanese encephalitis virus infection. Front Microbiol.

[34] Lei Y, Cao X, Xu W, Yang B, Xu Y, Zhou W, Dong S, Wu Q, Rahman K, Tyagi R (2021). Rv3722c promotes *Mycobacterium tuberculosis* survival in macrophages by interacting with TRAF3. Front Cell Infect Microbiol.

[35] Imaizumi T, Hashimoto S, Sato R, Umetsu H, Aizawa T, Watanabe S, Kawaguchi S, Matsumiya T, Seya K, Ding J, Tanaka H (2021). IFIT proteins are involved in CXCL10 expression in human glomerular endothelial cells treated with a toll-like receptor 3 agonist. Kidney Blood Press Res.

[36] Yan J, Zheng Y, Yuan P, Wang S, Han S, Yin J, Peng B, Li Z, Sun Y, He X, Liu W (2021). Novel host protein TBC1D16, a GTPase activating protein of Rab 5C, inhibits prototype foamy virus replication. Front Immunol.

[37] Su R, Shereen MA, Zeng X, Liang Y, Li W, Ruan Z, Li Y, Liu W, Liu Y, Wu K, Luo Z (2020). The TLR3/IRF1/Type III IFN axis facilitates antiviral responses against enterovirus infections in the intestine. MBio.

[38] Ferguson MC, Saul S, Fragkoudis R, Weisheit S, Cox J, Patabendige A, Sherwood K, Watson M, Merits A, Fazakerley JK (2015). Ability of the encephalitic arbovirus Semliki forest virus to cross the blood-brain barrier is determined by the charge of the E2 glycoprotein. J Virol.

[39] Tedelind S, Ericson LE, Karlsson JO, Nilsson M (2003). Interferon-gamma down-regulates claudin-1 and impairs the epithelial barrier function in primary cultured human thyrocytes. Eur J Endocrinol.

[40] Zhang L, Li Q, Ding X, Zhang B, Zhang Q, Qu X, Huo Y, Yang J, Wang S (2017). Antisense oligonucleotides targeting Raf-1 block Japanese encephalitis virus in vitro and in vivo. Nucleic Acid Ther.

[41] Perng YC, Lenschow DJ (2018). ISG15 in antiviral immunity and beyond. Nat Rev Microbiol.

[42] Espert L, Degols G, Gongora C, Blondel D, Williams BR, Silverman RH, Mechti N (2003). ISG20, a new interferon-induced RNase specific for single-stranded RNA, defines an alternative antiviral pathway against RNA genomic viruses. J Biol Chem.

[43] Jacquet S, Pontier D, Etienne L (2020). Rapid evolution of HERC6 and duplication of a chimeric HERC5/6 gene in rodents and bats suggest an overlooked role of HERCs in mammalian immunity. Front Immunol.

[44] Zhang M, Zhang MX, Zhang Q, Zhu GF, Yuan L, Zhang DE, Zhu Q, Yao J, Shu HB, Zhong B (2016). USP18 recruits USP20 to promote innate antiviral response through deubiquitinating STING/MITA. Cell Res.

[45] Das A, Dinh PX, Panda D, Pattnaik AK (2014). Interferon-inducible protein IFI35 negatively regulates RIG-I antiviral signaling and supports vesicular stomatitis virus replication. J Virol.

[46] Zhou P, Ma L, Rao Z, Li Y, Zheng H, He Q, Luo R (2021). Duck tembusu virus infection promotes the expression of duck interferon-induced protein 35 to counteract RIG-I antiviral signaling in duck embryo fibroblasts. Front Immunol.

[47] Wang K, Wang H, Lou W, Ma L, Li Y, Zhang N, Wang C, Li F, Awais M, Cao S (2018). IP-10 promotes blood-brain barrier damage by inducing tumor necrosis factor alpha production in Japanese encephalitis. Front Immunol.

[48] Mladinich MC, Conde JN, Schutt WR, Sohn SY, Mackow ER (2021). Blockade of autocrine CCL5 responses inhibits Zika virus persistence and spread in human brain microvascular endothelial cells. MBio.

[49] Steiner O, Coisne C, Cecchelli R, Boscacci R, Deutsch U, Engelhardt B, Lyck R (2010). Differential roles for endothelial ICAM-1, ICAM-2, and VCAM-1 in shear-resistant T cell arrest, polarization, and directed crawling on blood-brain barrier endothelium. J Immunol.

[50] Chen Z, Li G (2021). Immune response and blood-brain barrier dysfunction during viral neuroinvasion. Innate Immun.

[51] Honda K, Takaoka A, Taniguchi T (2006). Type I interferon [corrected] gene induction by the interferon regulatory factor family of transcription factors. Immunity.

[52] Odendall C, Kagan JC (2015). The unique regulation and functions of type III interferons in antiviral immunity. Curr Opin Virol.

[53] Daffis S, Samuel MA, Keller BC, Gale M, Diamond MS (2007). Cell-specific IRF-3 responses protect against West Nile virus infection by interferon-dependent and -independent mechanisms. PLoS Pathog.

[54] Wacher C, Müller M, Hofer MJ, Getts DR, Zabaras R, Ousman SS, Terenzi F, Sen GC, King NJ, Campbell IL (2007). Coordinated regulation and widespread cellular expression of interferon-stimulated genes (ISG) ISG-49, ISG-54, and ISG-56 in the central nervous system after infection with distinct viruses. J Virol.

[55] Emeny JM, Morgan MJ (1979). Regulation of the interferon system: evidence that Vero cells have a genetic defect in interferon production. J Gen Virol.

[56] Lokugamage KG, Hage A, de Vries M, Valero-Jimenez AM, Schindewolf C, Dittmann M, Rajsbaum R, Menachery VD (2020). Type I interferon susceptibility distinguishes SARS-CoV-2 from SARS-CoV. J Virol.

[57] Li Y, Li C, Xue P, Zhong B, Mao AP, Ran Y, Chen H, Wang YY, Yang F, Shu HB (2009). ISG56 is a negative-feedback regulator of virus-triggered signaling and cellular antiviral response. Proc Natl Acad Sci U S A.

[58] Zhang B, He Y, Xu Y, Mo F, Mi T, Shen QS, Li C, Li Y, Liu J, Wu Y (2018). Differential antiviral immunity to Japanese encephalitis virus in developing cortical organoids. Cell Death Dis.

[59] Sips GJ, Wilschut J, Smit JM (2012). Neuroinvasive flavivirus infections. Rev Med Virol.

[60] Greenwood J, Heasman SJ, Alvarez JI, Prat A, Lyck R, Engelhardt B (2011). Review: leucocyte-endothelial cell crosstalk at the blood-brain barrier: a prerequisite for successful immune cell entry to the brain. Neuropathol Appl Neurobiol.

[61] Kraus J, Voigt K, Schuller AM, Scholz M, Kim KS, Schilling M, Schäbitz WR, Oschmann P, Engelhardt B (2008). Interferon-beta stabilizes barrier characteristics of the blood-brain barrier in four different species in vitro. Mult Scler.

[62] Minagar A, Long A, Ma T, Jackson TH, Kelley RE, Ostanin DV, Sasaki M, Warren AC, Jawahar A, Cappell B, Alexander JS (2003). Interferon (IFN)-beta 1a and IFN-beta 1b block IFN-gamma-induced disintegration of endothelial junction integrity and barrier. Endothelium.

[63] Papa MP, Meuren LM, Coelho SVA, Lucas CGO, Mustafá YM, Lemos Matassoli F, Silveira PP, Frost PS, Pezzuto P, Ribeiro MR (2017). Zika virus infects, activates, and crosses brain microvascular endothelial cells, without barrier disruption. Front Microbiol.

[64] Johnson RT, Burke DS, Elwell M, Leake CJ, Nisalak A, Hoke CH, Lorsomrudee W (1985). Japanese encephalitis: immunocytochemical studies of viral antigen and inflammatory cells in fatal cases. Ann Neurol.

[65] Ramos C, Sánchez G, Pando RH, Baquera J, Hernández D, Mota J, Ramos J, Flores A, Llausás E (1998). Dengue virus in the brain of a fatal case of hemorrhagic dengue fever. J Neurovirol.

[66] Sun L, Wang X, Zhou Y, Zhou RH, Ho WZ, Li JL (2016). Exosomes contribute to the transmission of anti-HIV activity from TLR3-activated brain microvascular endothelial cells to macrophages. Antiviral Res.

[67] Han YW, Choi JY, Uyangaa E, Kim SB, Kim JH, Kim BS, Kim K, Eo SK (2014). Distinct dictation of Japanese encephalitis virus-induced neuroinflammation and lethality via triggering TLR3 and TLR4 signal pathways. PLoS Pathog.

[68] Kawai T, Akira S (2010). The role of pattern-recognition receptors in innate immunity: update on Toll-like receptors. Nat Immunol.

[69] Kato H, Takeuchi O, Sato S, Yoneyama M, Yamamoto M, Matsui K, Uematsu S, Jung A, Kawai T, Ishii KJ (2006). Differential roles of MDA5 and RIG-I helicases in the recognition of RNA viruses. Nature.

[70] Honda K, Yanai H, Negishi H, Asagiri M, Sato M, Mizutani T, Shimada N, Ohba Y, Takaoka A, Yoshida N, Taniguchi T (2005). IRF-7 is the master regulator of type-I interferon-dependent immune responses. Nature.

[71] Schneider WM, Chevillotte MD, Rice CM (2014). Interferon-stimulated genes: a complex web of host defenses. Annu Rev Immunol.

[72] Sooryanarain H, Rogers AJ, Cao D, Haac MER, Karpe YA, Meng XJ (2017). ISG15 modulates Type I interferon signaling and the antiviral response during hepatitis E virus replication. J Virol.

[73] Dai J, Pan W, Wang P (2011). ISG15 facilitates cellular antiviral response to dengue and west Nile virus infection in vitro. Virol J.

[74] Sadler AJ, Williams BR (2008). Interferon-inducible antiviral effectors. Nat Rev Immunol.

[75] Chen S, Zhang W, Wu Z, Zhang J, Wang M, Jia R, Zhu D, Liu M, Sun K, Yang Q (2017). Goose Mx and oasl play vital roles in the antiviral effects of type I, II, and III interferon against newly emerging avian flavivirus. Front Immunol.

[76] Horisberger MA (1995). Interferons, Mx genes, and resistance to influenza virus. Am J Respir Crit Care Med.

[77] Zhou J, Wang SQ, Wei JC, Zhang XM, Gao ZC, Liu K, Ma ZY, Chen PY, Zhou B (2017). Mx is not responsible for the antiviral activity of interferon-α against Japanese encephalitis virus. Viruses.

[78] Szretter KJ, Daniels BP, Cho H, Gainey MD, Yokoyama WM, Gale M, Virgin HW, Klein RS, Sen GC, Diamond MS (2012). 2'-O methylation of the viral mRNA cap by West Nile virus evades ifit1-dependent and -independent mechanisms of host restriction in vivo. PLoS Pathog.

[79] Chai B, Tian D, Zhou M, Tian B, Yuan Y, Sui B, Wang K, Pei J, Huang F, Wu Q (2021). Murine Ifit3 restricts the replication of Rabies virus both in vitro and in vivo. J Gen Virol.

[80] Cain MD, Salimi H, Gong Y, Yang L, Hamilton SL, Heffernan JR, Hou J, Miller MJ, Klein RS (2017). Virus entry and replication in the brain precedes blood-brain barrier disruption during intranasal alphavirus infection. J Neuroimmunol.

[81] Butchi NB, Hinton DR, Stohlman SA, Kapil P, Fensterl V, Sen GC, Bergmann CC (2014). Ifit2 deficiency results in uncontrolled neurotropic coronavirus replication and enhanced encephalitis via impaired alpha/beta interferon induction in macrophages. J Virol.

[82] Diamond MS, Farzan M (2013). The broad-spectrum antiviral functions of IFIT and IFITM proteins. Nat Rev Immunol.

[83] Zhou X, Michal JJ, Zhang L, Ding B, Lunney JK, Liu B, Jiang Z (2013). Interferon induced IFIT family genes in host antiviral defense. Int J Biol Sci.

[84] Lieberman NAP, Peddu V (2020). In vivo antiviral host transcriptional response to SARS-CoV-2 by viral load, sex, and age. PLoS Biol.

[85] Zhang L, Wang B, Li L, Qian DM, Yu H, Xue ML, Hu M, Song XX (2017). Antiviral effects of IFIT1 in human cytomegalovirus-infected fetal astrocytes. J Med Virol.

[86] Daffis S, Szretter KJ, Schriewer J, Li J, Youn S, Errett J, Lin TY, Schneller S, Zust R, Dong H (2010). 2'-O methylation of the viral mRNA cap evades host restriction by IFIT family members. Nature.

[87] Xu F, Song H, An B, Xiao Q, Cheng G, Tan G (2019). NF-κB-dependent IFIT3 Induction by HBx promotes hepatitis B virus replication. Front Microbiol.

[88] Pinto AK, Williams GD, Szretter KJ, White JP, Proença-Módena JL, Liu G, Olejnik J, Brien JD, Ebihara H, Mühlberger E (2015). Human and murine IFIT1 proteins do not restrict infection of negative-sense RNA viruses of the orthomyxoviridae, bunyaviridae, and filoviridae families. J Virol.

[89] Li C, Zhang W, Li Y, Guo L, Shu H, Liu Y (2012). ISG60 negatively regulates cell antiviral responses by disrupting the VISA-associated complexes. Wuhan University J Nat Sci.

[90] Patabendige A, Michael BD, Craig AG, Solomon T (2018). Brain microvascular endothelial-astrocyte cell responses following Japanese encephalitis virus infection in an in vitro human blood-brain barrier model. Mol Cell Neurosci.

[91] Daffis S, Samuel MA, Suthar MS, Gale M, Diamond MS (2008). Toll-like receptor 3 has a protective role against West Nile virus infection. J Virol.

[92] Diamond MS, Gale M (2012). Cell-intrinsic innate immune control of West Nile virus infection. Trends Immunol.

[93] Yong VW (2002). Differential mechanisms of action of interferon-beta and glatiramer aetate in MS. Neurology.

[94] Saha S, Sugumar P, Bhandari P, Rangarajan PN (2006). Identification of Japanese encephalitis virus-inducible genes in mouse brain and characterization of GARG39/IFIT2 as a microtubule-associated protein. J Gen Virol.

[95] Li Y, Zhang H, Zhu B, Ashraf U, Chen Z, Xu Q, Zhou D, Zheng B, Song Y, Chen H (2017). Microarray analysis identifies the potential role of long non-coding RNA in regulating neuroinflammation during Japanese encephalitis virus infection. Front Immunol.

[96] Agarwal S, Macfarlan TS, Sartor MA, Iwase S (2015). Sequencing of first-strand cDNA library reveals full-length transcriptomes. Nat Commun.

[97] Mäe MA, He L, Nordling S, Vazquez-Liebanas E, Nahar K, Jung B, Li X, Tan BC, Chin Foo J, Cazenave-Gassiot A (2021). Single-cell analysis of blood-brain barrier response to pericyte loss. Circ Res.

