# Supplementary material for: Type I/type III IFN and related factors regulate JEV infection and BBB endothelial integrity
Source: J Neuroinflammation. 2023 Sep 27;20:216. doi: 10.1186/s12974-023-02891-x (PMC10523659; doi:10.1186/s12974-023-02891-x)
Supplement: Supplementary file 1 — Additional file 1: Table S1. Primers employed for RT-qPCR in this study. Table S2. shRNA oligonucleotide sequences in this study. [file 12974_2023_2891_MOESM1_ESM.zip › Table S1. Primers employed for RT-qPCR in this study.docx]

**Table S1**

Primers employed for RT-qPCR in this study

| Gene | Forward (5′–3′) | Reverse (5′–3′) | Species |
| --- | --- | --- | --- |
| β-actin | AGCGGGAAATCGTGCGTGAC | GGAAGGAAGGCTGGAAGAGTG | Human |
| BST2 | CACACTGTGATGGCCCTAATG | GTCCGCGATTCTCACGCTT | Human |
| OAS3 | GCTTCAAGAGCTATGTGGACC | GGAAACGTGAGTCTCAGACCA | Human |
| TAP1 | CGCCTCACTGACTGGATTCTA | TCTGTTGGAAAAACTCCGTCTC | Human |
| TRIM22 | ACCAAACATTCCGCATAAACGA | AGGCGGTTCTCTCTTGTCTGA | Human |
| CCL2 | CCAGATGCAATCAATGCCC | ATGGTCTTGAAGATCACAGCT | Human |
| NPHS1 | CTGCCTGAAAACCTGACGGT | GACCTGGCACTCATACTCCG | Human |
| BNIP3L | TTGGATGCACAACATGAATCAGG | TCTTCTGACTGAGAGCTATGGTC | Human |
| PDK1 | GGATTGCCCATATCACGTCTTT | TCCCGTAACCCTCTAGGGAATA | Human |
| IFNβ | GACGCCGCATTGACCATCTA | TTGGCCTTCAGGTAATGCAGAA | Human |
| IFN-λ2,3 | CTGACGCTGAAGGTTCTGGAG | CGGAAGAGGTTGAAGGTGACAG | Human |
| IFIT1 | AGAAGCAGGCAATCACAGAAAA | CTGAAACCGACCATAGTGGAAAT | Human |
| IFIT2 | AAGCACCTCAAAGGGCAAAAC | AAGCACCTCAAAGGGCAAAAC | Human |
| IFIT3 | TCAGAAGTCTAGTCACTTGGGG | ACACCTTCGCCCTTTCATTTC | Human |
| IFIT5 | GGCCAAAATAAAGACGCCCTT | GACCAGGCTTCGTACTTCTTC | Human |
| IFITM1 | CCAAGGTCCACCGTGATTAAC | ACCAGTTCAAGAAGAGGGTGTT | Human |
| OAS1 | AGTTGACTGGCGGCTATAAAC | GTGCTTGACTAGGCGGATGAG | Human |
| OAS2 | AGGTGGCTCCTATGGACGG | TTTATCGAGGATGTCACGTTGG | Human |
| OASL | CCATTGTGCCTGCCTACAGAG | CTTCAGCTTAGTTGGCCGATG | Human |
| GBP4 | ATGGGTGAGAGAACTCTTCACG | TGCGGTATAGCCCTACAATGG | Human |
| ISG15 | CGCAGATCACCCAGAAGATCG | TTCGTCGCATTTGTCCACCA | Human |
| USP18 | CCTGAGGCAAATCTGTCAGTC | CGAACACCTGAATCAAGGAGTTA | Human |
| IRF7 | CCCACGCTATACCATCTACCT | GATGTCGTCATAGAGGCTGTTG | Human |
| IFIH1 | TCACAAGTTGATGGTCCTCAAGT | CTGATGAGTTATTCTCCATGCCC | Human |
| IFI35 | AACAAAAGGAGCACACGATCA | CTCCGTTCCTAGTCTTGCCAA | Human |
| IFI44 | TTTTCGATGCGAAGATTCACTGG | CCTGATGCGTTACATGCCCTT | Human |
| CCL5 | ATATTCCTCGGACACCACAC | GTGACAAAGACGACTGCTG | Human |
| CXCL10 | CCTGCTGGGTCTGAGTGGGA | GATAGGCT CGCAGGGATGAT | Human |
| DDX58 | TGCGAATCAGATCCCAGTGTA | TGCCTGTAACTCTATACCCATGT | Human |
| TLR3 | CAAACACAAGCATTCGGAATCTG | AAGGAATCGTTACCAACCACATT | Human |
| JEV-C | GGCTCTTATCACGTTCTTCAAGTTT | TGCTTTCCATCGGCCYAAAA |  |
