# Supplementary material for: Type I/type III IFN and related factors regulate JEV infection and BBB endothelial integrity
Source: J Neuroinflammation. 2023 Sep 27;20:216. doi: 10.1186/s12974-023-02891-x (PMC10523659; doi:10.1186/s12974-023-02891-x)
Supplement: Supplementary file 1 — Additional file 1: Table S1. Primers employed for RT-qPCR in this study. Table S2. shRNA oligonucleotide sequences in this study. [file 12974_2023_2891_MOESM1_ESM.zip › Table S2 shRNA oligonuleotides sequences in this study.docx]

**Table S2**

shRNA oligonuleotides sequences in this study

| shRNA ID | Target sequence (5'-3') |  |
| --- | --- | --- |
| shNC | CAACAAGATGAAGAGCACCAA |  |
| shTLR3 | ACTTAAATGTGGTTGGTAA |  |
| shDDX58 | TGCAATCTTGTCATCCTTTAT |  |
| shIFIH1 | CCAACAAAGAAGCAGTGTATA |  |
| shIFIT1 | GGATAAAGCTCTTGAGTTA |  |
| shIFIT2 | GCCAAATCCTTCATGTAATATT |  |
| shIFIT3 | GCTATGGACTATTCGAATAAA |  |
| shIFIT5 | GAACCCAGATAACAGCTATAT |  |
