# Supplementary figures and images for: Type I/type III IFN and related factors regulate JEV infection and BBB endothelial integrity
Source: J Neuroinflammation. 2023 Sep 27;20:216. doi: 10.1186/s12974-023-02891-x (PMC10523659; doi:10.1186/s12974-023-02891-x)

**A**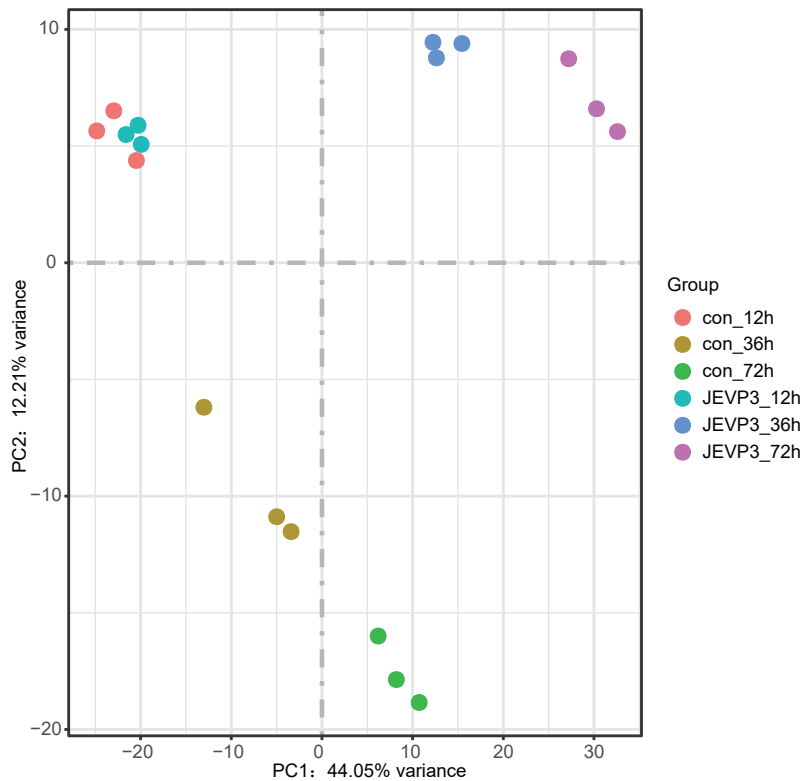

Supplement: Supplementary file 2 — Additional file 2: Fig. S1. Principal component analysis (PCA) of JEV-infected hBMECs at 12, 36, and 72 hpi. (A) Note: PC1 illustrates the differences among JEV-infected samples; PC2 shows differences between mock-infected and JEV-infected samples at different time points, including 12, 36, and 72 hpi. [file 12974_2023_2891_MOESM2_ESM.pdf]

**A**

JEV VS Mock 12h.volcano

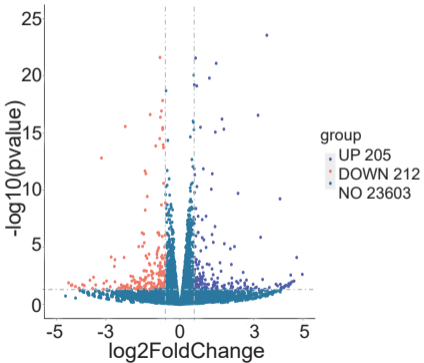**B**

JEV VS Mock 36h.volcano

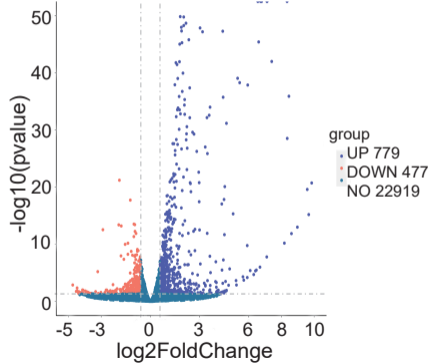**C**

JEV VS Mock 72h.volcano

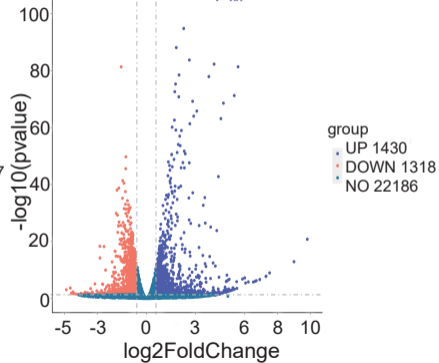

Supplement: Supplementary file 4 — Additional file 4: Fig. S2. Volcano plot of global DEGs at different time points. V P3 vs. Mock 12 h (A), JEV P3 vs. Mock 36 h (B), JEV P3 vs. Mock 72 h (C). Purple dots (right) and red dots (left) represent significantly upregulated and downregulated genes, respectively. Blue dots (middle) represent insignificantly expressed genes. Fig. S3. GO and KEGG enrichment statistics. The top 30 GO terms were clustered into 3 categories, including cellular component (CC), molecular function (MF), and biological process (BP), mock VS JEV 12 h (A), mock VS JEV 36 h (C), and mock VS JEV 72 h (E). Top 20 Kyoto Encyclopedia of Genes and Genomes (KEGG) pathways in JEV-infected hBMECs, mock vs JEV 12 h (B), mock vs JEV 36 h (D), and mock vs JEV 72 h (F). [file 12974_2023_2891_MOESM4_ESM.zip › Fig. S2. Volcano plot of global DEGs at different time points.pdf]

**A**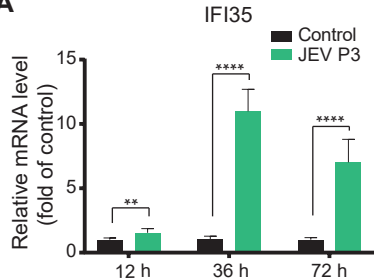**B**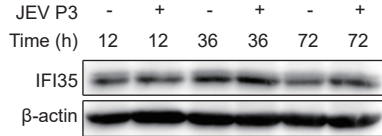**C**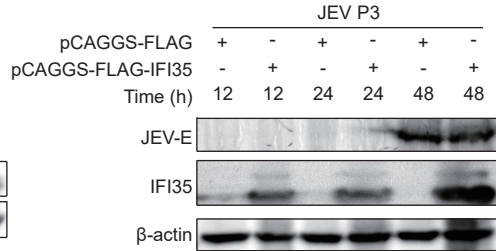

Supplement: Supplementary file 6 — Additional file 6: Fig. S4. JEV infection induced the upregulation of IFI35 in hBMECs. hBMECs were infected with JEV P3 for the indicated time, and the expression of IFI35 at the mRNA level (A) and protein level (B) was measured by performing RT-qPCR and Western blotting, respectively. (C) HEK293T cells were transfected with an empty vector (vector) or IFI35 (Vector-IFI35) as indicated, and the expression of IFI35 and JEV-E at the protein level was measured by Western blotting. Data are represented as the mean values ± SEMs from three independent experiments. **p < 0.01; ****p < 0.0001. Fig. S5. JEV infection triggers the induction of ISGs in hBMECs, Related to Table 2. The hBMECs were infected with JEV P3 for the indicated time, and mRNA levels of IFN-λ2,3, IFITM1, ISG15, OAS1, OAS2, OASL, GBP4, CXCL10, USP18, IFI44, and CCL5 in these cells were quantified by RT-qPCR (A), and the protein levels of USP18, IFITM1, and ISG15 were measured by Western blotting (B). Fig. S6. JEV infection induced IRF activation in hBMECs. The hBMECs were infected with JEV P3 for the indicated time, and the expression of IRF7 and IRF1 at the mRNA level (A) and IRF7, IRF1, and IRF3/p-IRF3 at the protein level (B) were measured by performing RT-qPCR and Western blotting, respectively. (C) Cells were infected with JEV P3 for 36 h, and the protein levels of IRF7, IRF1, and IRF3/p-IRF3 were measured in shCTL, shTLR3, shRIG-I, and shMDA5 hBMECs, representing significantly upregulated and downregulated genes, respectively. Blue dots (middle) represent insignificantly expressed genes. Data are represented as the mean values ± SEMs from three independent experiments. ** p < 0.01; ****, p < 0.0001. Fig. S7. IFN-β/IFN-λ1 treatment induced IFIT mRNA expression in hBMECs. (A, C) hBMECs were treated with distinct concentrations of rhIFN-β/rhIFN-λ1 protein, and IFIT mRNA expression was measured. (B, D) Cells were treated with 10 ng/ml rhIFN-β (B) or 100 ng/ml rhIFN-λ1 protein (D) for different time poin [file 12974_2023_2891_MOESM6_ESM.zip › Fig. S4.pdf]

**A**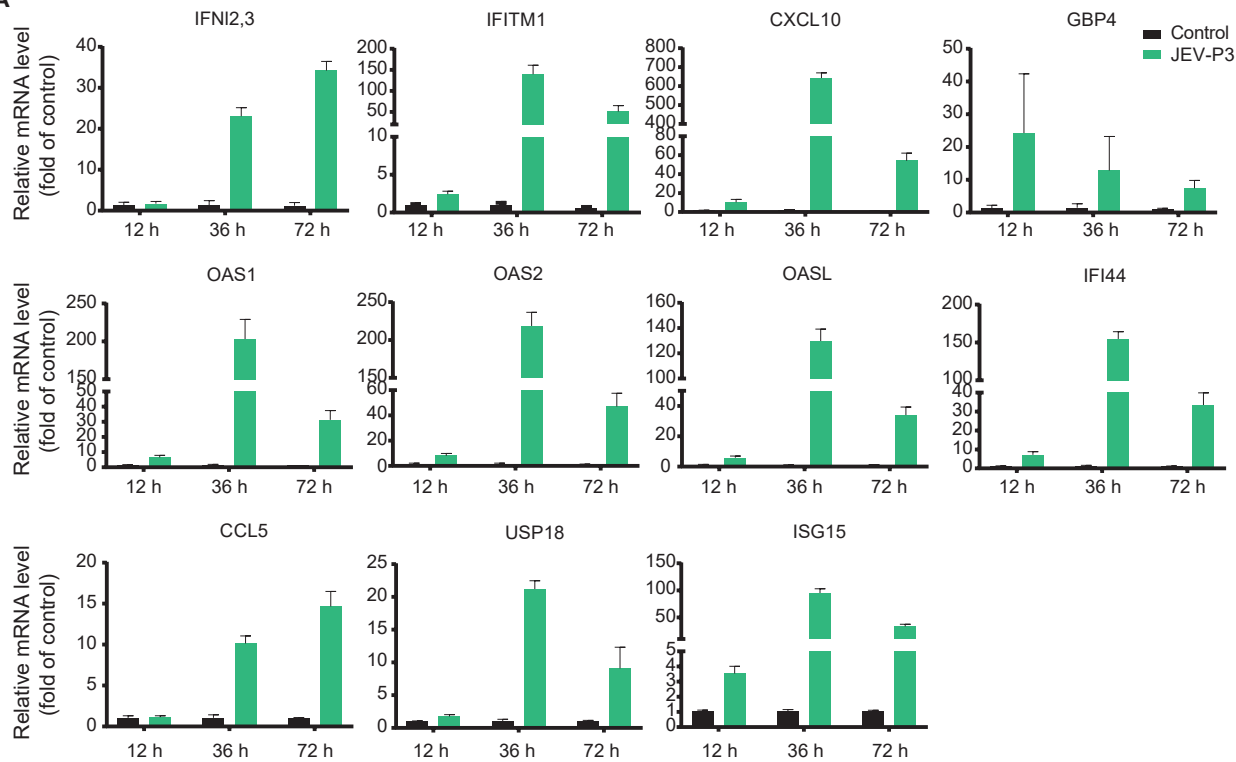**B**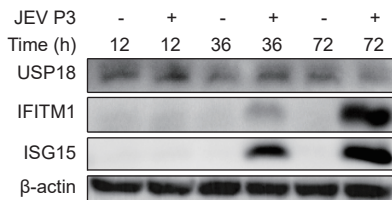

Supplement: Supplementary file 6 — Additional file 6: Fig. S4. JEV infection induced the upregulation of IFI35 in hBMECs. hBMECs were infected with JEV P3 for the indicated time, and the expression of IFI35 at the mRNA level (A) and protein level (B) was measured by performing RT-qPCR and Western blotting, respectively. (C) HEK293T cells were transfected with an empty vector (vector) or IFI35 (Vector-IFI35) as indicated, and the expression of IFI35 and JEV-E at the protein level was measured by Western blotting. Data are represented as the mean values ± SEMs from three independent experiments. **p < 0.01; ****p < 0.0001. Fig. S5. JEV infection triggers the induction of ISGs in hBMECs, Related to Table 2. The hBMECs were infected with JEV P3 for the indicated time, and mRNA levels of IFN-λ2,3, IFITM1, ISG15, OAS1, OAS2, OASL, GBP4, CXCL10, USP18, IFI44, and CCL5 in these cells were quantified by RT-qPCR (A), and the protein levels of USP18, IFITM1, and ISG15 were measured by Western blotting (B). Fig. S6. JEV infection induced IRF activation in hBMECs. The hBMECs were infected with JEV P3 for the indicated time, and the expression of IRF7 and IRF1 at the mRNA level (A) and IRF7, IRF1, and IRF3/p-IRF3 at the protein level (B) were measured by performing RT-qPCR and Western blotting, respectively. (C) Cells were infected with JEV P3 for 36 h, and the protein levels of IRF7, IRF1, and IRF3/p-IRF3 were measured in shCTL, shTLR3, shRIG-I, and shMDA5 hBMECs, representing significantly upregulated and downregulated genes, respectively. Blue dots (middle) represent insignificantly expressed genes. Data are represented as the mean values ± SEMs from three independent experiments. ** p < 0.01; ****, p < 0.0001. Fig. S7. IFN-β/IFN-λ1 treatment induced IFIT mRNA expression in hBMECs. (A, C) hBMECs were treated with distinct concentrations of rhIFN-β/rhIFN-λ1 protein, and IFIT mRNA expression was measured. (B, D) Cells were treated with 10 ng/ml rhIFN-β (B) or 100 ng/ml rhIFN-λ1 protein (D) for different time poin [file 12974_2023_2891_MOESM6_ESM.zip › Fig. S5.pdf]

**A**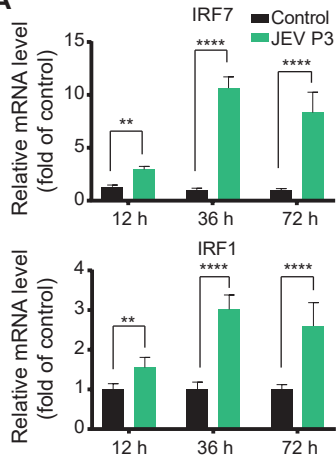**B**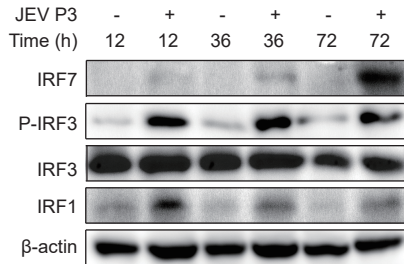**C**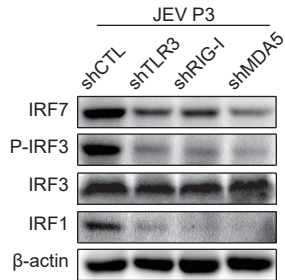

Supplement: Supplementary file 6 — Additional file 6: Fig. S4. JEV infection induced the upregulation of IFI35 in hBMECs. hBMECs were infected with JEV P3 for the indicated time, and the expression of IFI35 at the mRNA level (A) and protein level (B) was measured by performing RT-qPCR and Western blotting, respectively. (C) HEK293T cells were transfected with an empty vector (vector) or IFI35 (Vector-IFI35) as indicated, and the expression of IFI35 and JEV-E at the protein level was measured by Western blotting. Data are represented as the mean values ± SEMs from three independent experiments. **p < 0.01; ****p < 0.0001. Fig. S5. JEV infection triggers the induction of ISGs in hBMECs, Related to Table 2. The hBMECs were infected with JEV P3 for the indicated time, and mRNA levels of IFN-λ2,3, IFITM1, ISG15, OAS1, OAS2, OASL, GBP4, CXCL10, USP18, IFI44, and CCL5 in these cells were quantified by RT-qPCR (A), and the protein levels of USP18, IFITM1, and ISG15 were measured by Western blotting (B). Fig. S6. JEV infection induced IRF activation in hBMECs. The hBMECs were infected with JEV P3 for the indicated time, and the expression of IRF7 and IRF1 at the mRNA level (A) and IRF7, IRF1, and IRF3/p-IRF3 at the protein level (B) were measured by performing RT-qPCR and Western blotting, respectively. (C) Cells were infected with JEV P3 for 36 h, and the protein levels of IRF7, IRF1, and IRF3/p-IRF3 were measured in shCTL, shTLR3, shRIG-I, and shMDA5 hBMECs, representing significantly upregulated and downregulated genes, respectively. Blue dots (middle) represent insignificantly expressed genes. Data are represented as the mean values ± SEMs from three independent experiments. ** p < 0.01; ****, p < 0.0001. Fig. S7. IFN-β/IFN-λ1 treatment induced IFIT mRNA expression in hBMECs. (A, C) hBMECs were treated with distinct concentrations of rhIFN-β/rhIFN-λ1 protein, and IFIT mRNA expression was measured. (B, D) Cells were treated with 10 ng/ml rhIFN-β (B) or 100 ng/ml rhIFN-λ1 protein (D) for different time poin [file 12974_2023_2891_MOESM6_ESM.zip › Fig. S6.pdf]

**A**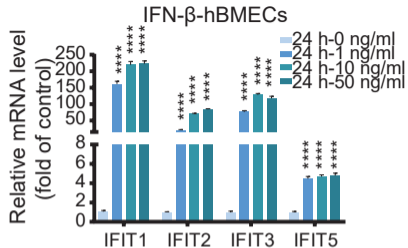**B**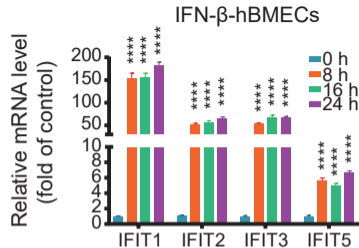**C**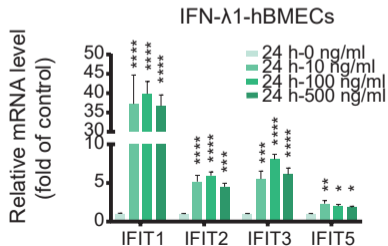**D**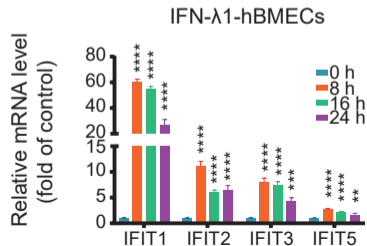

Supplement: Supplementary file 6 — Additional file 6: Fig. S4. JEV infection induced the upregulation of IFI35 in hBMECs. hBMECs were infected with JEV P3 for the indicated time, and the expression of IFI35 at the mRNA level (A) and protein level (B) was measured by performing RT-qPCR and Western blotting, respectively. (C) HEK293T cells were transfected with an empty vector (vector) or IFI35 (Vector-IFI35) as indicated, and the expression of IFI35 and JEV-E at the protein level was measured by Western blotting. Data are represented as the mean values ± SEMs from three independent experiments. **p < 0.01; ****p < 0.0001. Fig. S5. JEV infection triggers the induction of ISGs in hBMECs, Related to Table 2. The hBMECs were infected with JEV P3 for the indicated time, and mRNA levels of IFN-λ2,3, IFITM1, ISG15, OAS1, OAS2, OASL, GBP4, CXCL10, USP18, IFI44, and CCL5 in these cells were quantified by RT-qPCR (A), and the protein levels of USP18, IFITM1, and ISG15 were measured by Western blotting (B). Fig. S6. JEV infection induced IRF activation in hBMECs. The hBMECs were infected with JEV P3 for the indicated time, and the expression of IRF7 and IRF1 at the mRNA level (A) and IRF7, IRF1, and IRF3/p-IRF3 at the protein level (B) were measured by performing RT-qPCR and Western blotting, respectively. (C) Cells were infected with JEV P3 for 36 h, and the protein levels of IRF7, IRF1, and IRF3/p-IRF3 were measured in shCTL, shTLR3, shRIG-I, and shMDA5 hBMECs, representing significantly upregulated and downregulated genes, respectively. Blue dots (middle) represent insignificantly expressed genes. Data are represented as the mean values ± SEMs from three independent experiments. ** p < 0.01; ****, p < 0.0001. Fig. S7. IFN-β/IFN-λ1 treatment induced IFIT mRNA expression in hBMECs. (A, C) hBMECs were treated with distinct concentrations of rhIFN-β/rhIFN-λ1 protein, and IFIT mRNA expression was measured. (B, D) Cells were treated with 10 ng/ml rhIFN-β (B) or 100 ng/ml rhIFN-λ1 protein (D) for different time poin [file 12974_2023_2891_MOESM6_ESM.zip › Fig. S7.pdf]
