# Supplementary material for: Type I/type III IFN and related factors regulate JEV infection and BBB endothelial integrity
Source: J Neuroinflammation. 2023 Sep 27;20:216. doi: 10.1186/s12974-023-02891-x (PMC10523659; doi:10.1186/s12974-023-02891-x)
Supplement: Supplementary file 4 — Additional file 4: Fig. S2. Volcano plot of global DEGs at different time points. V P3 vs. Mock 12 h (A), JEV P3 vs. Mock 36 h (B), JEV P3 vs. Mock 72 h (C). Purple dots (right) and red dots (left) represent significantly upregulated and downregulated genes, respectively. Blue dots (middle) represent insignificantly expressed genes. Fig. S3. GO and KEGG enrichment statistics. The top 30 GO terms were clustered into 3 categories, including cellular component (CC), molecular function (MF), and biological process (BP), mock VS JEV 12 h (A), mock VS JEV 36 h (C), and mock VS JEV 72 h (E). Top 20 Kyoto Encyclopedia of Genes and Genomes (KEGG) pathways in JEV-infected hBMECs, mock vs JEV 12 h (B), mock vs JEV 36 h (D), and mock vs JEV 72 h (F). [file 12974_2023_2891_MOESM4_ESM.zip › Fig. S3. GO and KEGG enrichment statistics.pdf]

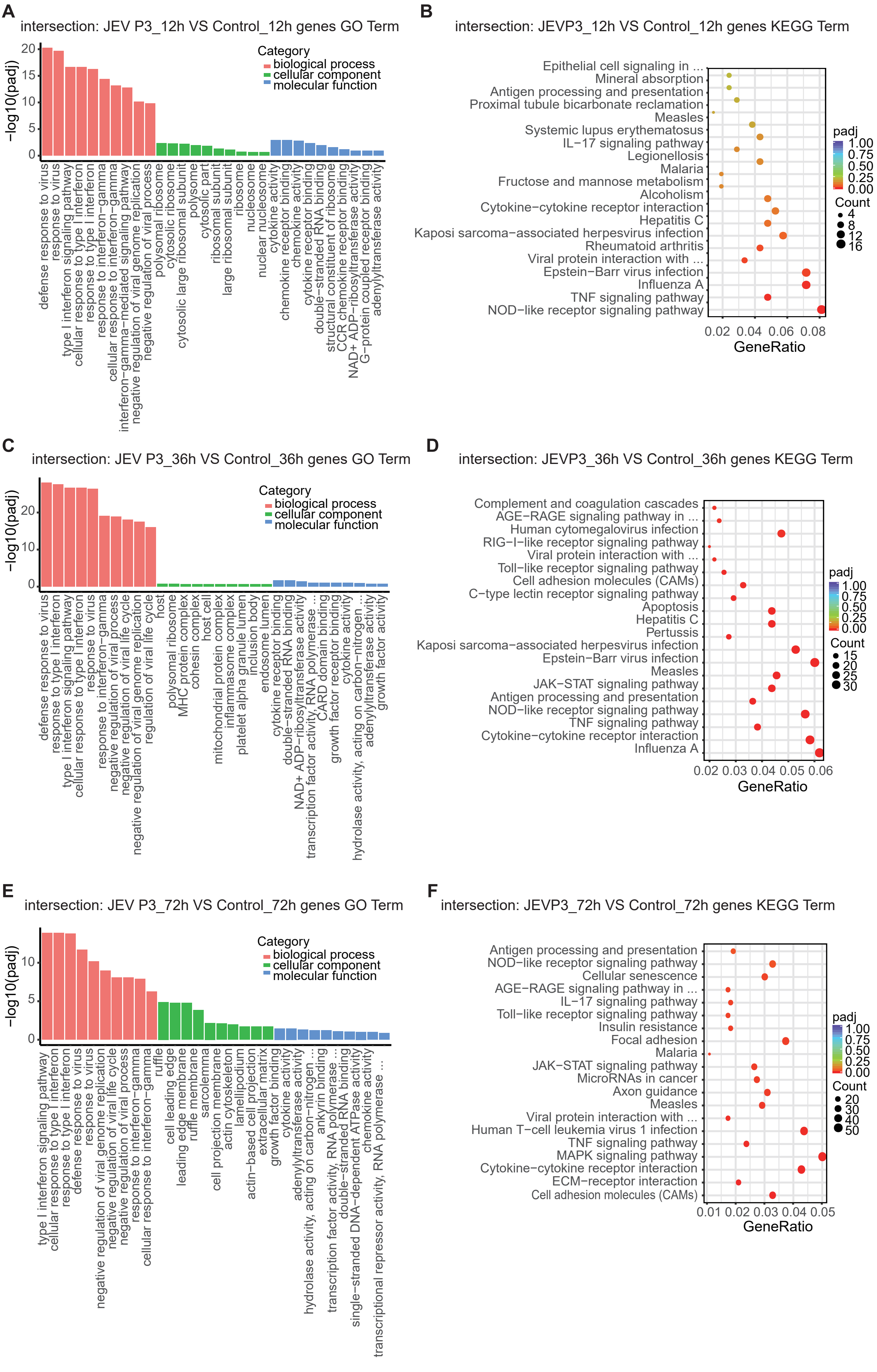
A
intersection: JEV P3_12h VS Control_12h genes GO Term
-log10(padj)
0
5
10
15
20
Category
biological process
cellular component
molecular function
defense response to virus
response to virus
type I interferon signaling pathway
cellular response to type I interferon
response to type I interferon
response to interferon−gamma
cellular response to interferon−gamma
interferon−gamma−mediated signaling pathway
negative regulation of viral genome replication
negative regulation of viral process
polysomal ribosome
cytosolic ribosome
cytosolic large ribosomal subunit
polysome
cytosolic part
ribosomal subunit
large ribosomal subunit
ribosome
nucleosome
nuclear nucleosome
cytokine activity
chemokine receptor binding
chemokine activity
cytokine receptor binding
double−stranded RNA binding
structural constituent of ribosome
CCR chemokine receptor binding
NAD+ ADP−ribosyltransferase activity
G−protein coupled receptor binding
adenylyltransferase activity
B
intersection: JEVP3_12h VS Control_12h genes KEGG Term
Epithelial cell signaling in ...
Mineral absorption
Antigen processing and presentation
Proximal tubule bicarbonate reclamation
Measles
Systemic lupus erythematosus
IL−17 signaling pathway
Legionellosis
Malaria
Fructose and mannose metabolism
Alcoholism
Cytokine−cytokine receptor interaction
Hepatitis C
Kaposi sarcoma−associated herpesvirus infection
Rheumatoid arthritis
Viral protein interaction with ...
Epstein−Barr virus infection
Influenza A
TNF signaling pathway
NOD−like receptor signaling pathway
0.02 0.04 0.06 0.08
GeneRatio
padj
1.00
0.75
0.50
0.25
0.00
Count
4
8
12
16
C
intersection: JEV P3_36h VS Control_36h genes GO Term
-log10(padj)
0
10
20
Category
biological process
cellular component
molecular function
defense response to virus
response to type I interferon
type I interferon signaling pathway
cellular response to type I interferon
response to virus
response to interferon−gamma
negative regulation of viral process
negative regulation of viral life cycle
negative regulation of viral genome replication
regulation of viral life cycle
host
polysomal ribosome
MHC protein complex
cohesin complex
host cell
mitochondrial protein complex
inflammasome complex
platelet alpha granule lumen
inclusion body
endosome lumen
cytokine receptor binding
double−stranded RNA binding
NAD+ ADP−ribosyltransferase activity
transcription factor activity, RNA polymerase ...
CARD domain binding
growth factor receptor binding
cytokine activity
hydrolase activity, acting on carbon−nitrogen ...
adenylyltransferase activity
growth factor activity
D
intersection: JEVP3_36h VS Control_36h genes KEGG Term
Complement and coagulation cascades
AGE−RAGE signaling pathway in ...
Human cytomegalovirus infection
RIG−I−like receptor signaling pathway
Viral protein interaction with ...
Toll−like receptor signaling pathway
Cell adhesion molecules (CAMs)
C−type lectin receptor signaling pathway
Apoptosis
Hepatitis C
Pertussis
Kaposi sarcoma−associated herpesvirus infection
Epstein−Barr virus infection
Measles
JAK−STAT signaling pathway
Antigen processing and presentation
NOD−like receptor signaling pathway
TNF signaling pathway
Cytokine−cytokine receptor interaction
Influenza A
0.02 0.03 0.04 0.05 0.06
GeneRatio
padj
1.00
0.75
0.50
0.25
0.00
Count
15
20
25
30
E
intersection: JEV P3_72h VS Control_72h genes GO Term
-log10(padj)
0
5
10
Category
biological process
cellular component
molecular function
type I interferon signaling pathway
cellular response to type I interferon
response to type I interferon
defense response to virus
response to virus
negative regulation of viral genome replication
negative regulation of viral life cycle
negative regulation of viral process
response to interferon−gamma
cellular response to interferon−gamma
ruffle
cell leading edge
leading edge membrane
ruffle membrane
sarcolemma
cell projection membrane
actin cytoskeleton
lamellipodium
actin−based cell projection
extracellular matrix
growth factor binding
cytokine activity
adenylyltransferase activity
hydrolase activity, acting on carbon−nitrogen ...
ankyrin binding
transcription factor activity, RNA polymerase ...
double−stranded RNA binding
single−stranded DNA−dependent ATPase activity
chemokine activity
transcriptional repressor activity, RNA polymerase ...
F
intersection: JEVP3_72h VS Control_72h genes KEGG Term
Antigen processing and presentation
NOD−like receptor signaling pathway
Cellular senescence
AGE−RAGE signaling pathway in ...
IL−17 signaling pathway
Toll−like receptor signaling pathway
Insulin resistance
Focal adhesion
Malaria
JAK−STAT signaling pathway
MicroRNAs in cancer
Axon guidance
Measles
Viral protein interaction with ...
Human T−cell leukemia virus 1 infection
TNF signaling pathway
MAPK signaling pathway
Cytokine−cytokine receptor interaction
ECM−receptor interaction
Cell adhesion molecules (CAMs)
0.01 0.02 0.03 0.04 0.05
GeneRatio
padj
1.00
0.75
0.50
0.25
0.00
Count
20
30
40
50
