# Supplementary material for: Type I/type III IFN and related factors regulate JEV infection and BBB endothelial integrity
Source: J Neuroinflammation. 2023 Sep 27;20:216. doi: 10.1186/s12974-023-02891-x (PMC10523659; doi:10.1186/s12974-023-02891-x)
Supplement: Supplementary file 5 — Additional file 5: Table S4. Information on the GO pathways of the DEGs in JEV-infected hBMECs at 12, 36, and 72 hpi. Table S5. Information on the KEGG pathways of the DEGs in JEV-infected hBMECs at 12, 36, and 72 hpi. Table S6. Global transcriptional response of JEV-infected hBMECs. [file 12974_2023_2891_MOESM5_ESM.zip › Table S6. Global transcriptional reponse of JEV-infected hBMECs.docx]

**Table S6 Critical DEGs in JEV-infected hBMECs**

| Category and gene | Fold induction versus controls at: | | | Confirmed by  RT-qPCR |
| --- | --- | --- | --- | --- |
|  | 12 hpi | 36 hpi | 72 hpi |  |
| IFN |  |  |  |  |
| IFN-β |  | 9.67 | 7.52 | √ |
| IFN-λ1 | 4.57 | 8.46 | 11.4 | √ |
| IFN-λ2 | 5.25 | 9.58 | 10.84 | √ |
| IFN-λ3 |  | 11.57 | 9.82 | √ |
| ISGs |  |  |  |  |
| IFIT1 | 2.63 | 7.12 | 4.47 | √ |
| IFIT2 | 1.79 | 6.73 | 3.95 | √ |
| IFIT3 | 1.71 | 5.54 | 3.93 | √ |
| IFIT5 |  | 2.2 | 1.49 | √ |
| IFITM1 | 1.36 | 6.56 | 5.39 | √ |
| MX1 |  | 2.41 | 1.66 |  |
| OAS1 | 5.21 | 8.08 | 4.28 | √ |
| OAS2 | 4.07 | 8.34 | 5.53 | √ |
| OAS3 | 1.01 | 3.33 | 2.16 | √ |
| OASL | 3.18 | 7.12 | 5.15 | √ |
| BST2 | 2.57 | 7.55 | 5.79 | √ |
| GBP4 | 0.76 | 3.05 | 1.87 | √ |
| ISG15 | 2.04 | 6.03 | 4.32 | √ |
| USP18 | 0.63 | 3.60 | 2.69 | √ |
| HERC5 | 0.91 | 4.41 | 3.59 |  |
| IFI35 |  | 3.25 | 1.80 | √ |
| IFI44 | 3.28 | 6.34 | 4.87 | √ |
| PRRs, IRFs and related |  |  |  |  |
| TLR3 |  | 1.33 | 0.67 | √ |
| RIG-I |  | 2.40 | 1.76 | √ |
| MDA5 | 0.75 | 3.18 | 2.23 | √ |
| IRF1 | 0.69 | 1.22 | 0.71 | √ |
| IRF7 | 1.43 | 3.02 | 2.28 | √ |
| STAT1 | 0.77 | 2.67 | 1.98 | √ |
| Chemokines and Inflammatory cytokines |  |  |  |  |
| CCL2/MCP1 | 0.75 | -1.01 | -2.54 | √ |
| CCL5/RANTES | 1.81 | 4.71 | 3.36 | √ |
| CXCL10 |  | 8.97 | 5.86 | √ |
| IL-1β |  |  | -1.03 |  |
| IL-6 |  |  | -0.8 |  |
| IL-12A |  | 1.60 | 1.05 |  |
| Cell adhesion molecules related |  |  |  |  |
| ICAM1 |  | 0.64 |  |  |
| ICAM2 |  |  | 2.06 |  |
| ICAM5 |  |  | 0.70 |  |
| VCAM1 |  |  | -0.78 |  |
