# Supplementary material for: Type I/type III IFN and related factors regulate JEV infection and BBB endothelial integrity
Source: J Neuroinflammation. 2023 Sep 27;20:216. doi: 10.1186/s12974-023-02891-x (PMC10523659; doi:10.1186/s12974-023-02891-x)
Supplement: Supplementary file 7 — Additional file 7: Table S7. List of antibodies used in this study. [file 12974_2023_2891_MOESM7_ESM.docx]

**Table S8**

List of antibodies used in this study

| Antibodies | company | catalog |
| --- | --- | --- |
| p-STAT1 | Abmart | T55702S |
| STAT1 |  | T55227S |
| IFI35 |  | PK60547 |
| IFIT2 | ABclonal | A13760 |
| TLR3 |  | A11778 |
| IRF7 |  | A0159 |
| IRF3 |  | A2172 |
| USP18 |  | A16739 |
| ISG15 |  | A1182 |
| JAK1 | Abcam | ab133666 |
| JAK2 |  | ab108596 |
| p-JAK1 |  | ab138005 |
| p-JAK2 |  | ab32101 |
| MDA5 | CST | 5321S |
| p-IRF3 |  | 4947S |
| RIG-I | Proteintech | 20566-1-AP |
| IRF1 |  | 11335-1-AP |
| IFIT1 |  | 23247-1-AP |
| IFIT3 |  | 15201-1-AP |
| IFIT5 |  | 13378-1-AP |
| IFITM1 |  | 60074-1-Ig |
| β-actin |  | 20566-1-AP |
| FLAG |  | 66008-4-Ig |
